# Semiconducting THO‐C_3_N Monolayers for Ultrahigh Anisotropic Carrier Mobility

**DOI:** 10.1002/advs.202519861

**Published:** 2026-01-15

**Authors:** Rui Tan, Xueqing Chen, Jifeng Luo, Zhe Xue, Zehou Li, Xiaolin Wei, Zhenkun Tang, Gaokuo Zhong

**Affiliations:** ^1^ The Key Laboratory of Micro‐nano Energy Materials and Application Technologiescollege of Physics and Electronic Engineering University of Hunan Province Hengyang Normal University Hengyang P. R. China; ^2^ Changsha Semiconductor Technology and Application Innovation Research Institute College of Semiconductors (College of Integrated Circuits) Hunan University Changsha P. R. China; ^3^ School of Materials Science and Engineering Collaborative Innovation Center of Ministry of Education and Shanxi Province For High‐performance Al/Mg Alloy Materials North University of China Taiyuan P. R. China

**Keywords:** 2D carbon nitrides, biphenylene‐based structures, carrier mobility, doping engineering

## Abstract

Biphenylene‐based structures with semiconducting characteristics hold great promise for nanoelectronics owing to their intrinsically anisotropic charge transport. Yet, achieving semiconducting behavior in these systems remains challenging due to their inherent metallic nature. Here, we propose a precise site‐specific N‐doping strategy that drives a secondary electronic transition in net W, enabling the electronic properties transition from metal to Dirac semimetal and ultimately to semiconductor. This transition is governed by the synergistic interplay of structural distortion, band‐filling, on‐site energy differences, and symmetry. The optimized THO‐C_3_N‐2 and THO‐C_3_N‐3 semiconductors exhibit high carrier mobilities (exceeding 10^3^ cm^2^ V^−1^ s^−1^) and pronounced mobility anisotropy, with THO‐C_3_N‐2 achieving the highest electron mobility anisotropy ratio (2061.22) among reported 2D carbon nitride systems. This work not only establishes an effective band engineering paradigm for biphenylene‐based materials but also offers promising candidates for directionally tailored nanoelectronic devices.

## Graphical abstract

A precise site‐specific N‐doping strategy that drives a secondary electronic transition in net W is proposed, enabling the electronic properties transition from metal to Dirac semimetal and ultimately to semiconductor. The obtained THO‐C_3_N‐2 and THO‐C_3_N‐3 semiconductors exhibit high carrier mobilities and pronounced mobility anisotropy, with THO‐C_3_N‐2 achieving the highest electron mobility anisotropy ratio among reported 2D carbon nitrides.

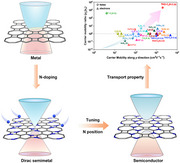

## 1 Introduction

The remarkable versatility of carbon atoms in forming diverse structural configurations across multiple dimensions has stimulated extensive research into novel carbon allotropes composed of multi‐membered rings and polygonal networks. Representative examples include biphenylene [1], net W [2], and net Y [3], which are constructed from four‐, six‐, and eight‐membered *sp* ^2^‐hybridized carbon rings. These allotropes exhibit mechanical, electronic, and thermal properties fundamentally distinct from those of honeycomb graphene [4], and hold promise for applications in fields such as 2D sensing devices [5], superconductivity [6], and metal‐ion batteries [7, 8]. Unlike the hexagonal symmetry of graphene, biphenylene‐based carbon allotropes typically adopt orthorhombic structures arising from the coexistence of four‐, six‐, and eight‐membered rings. This distinctive atomic arrangement leads to pronounced anisotropic properties, including electrical transport [5, 9], thermal conductivity [10], and mechanical responses [7], as well as intriguing phenomena such as negative differential resistance and negative thermal expansion [5, 11]. Such anisotropy renders these materials particularly attractive for direction‐dependent device applications. However, despite their exciting potential, all biphenylene‐based carbon allotropes reported to date exhibit metallic behavior [7], which severely restricts their utility in nanoelectronics and optoelectronics. Therefore, developing effective strategies to tailor their electronic structures and induce bandgap opening remains a critical challenge in advancing this emerging field.

**FIGURE 1:**
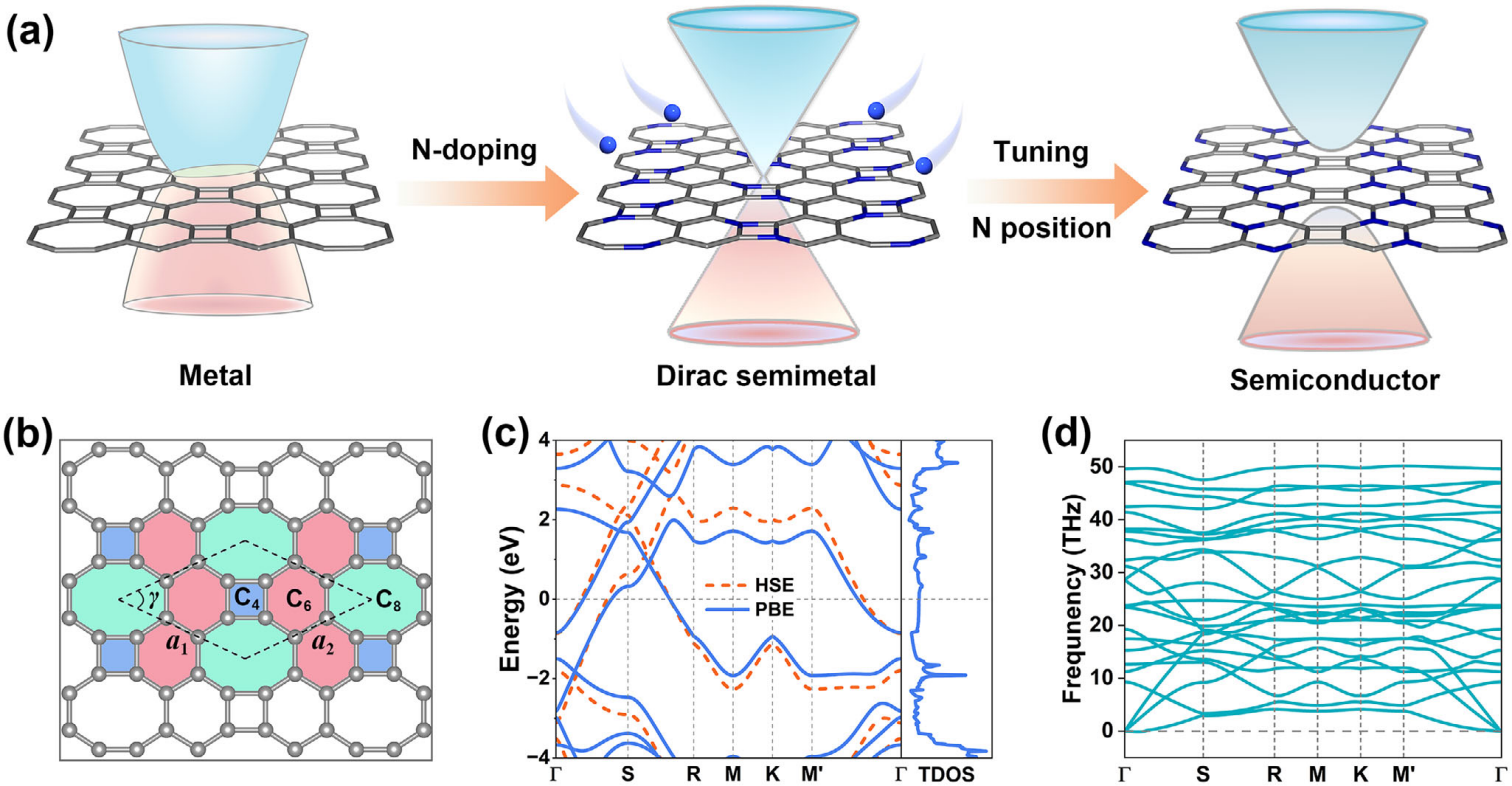
(a) Schematic illustration of band structure modulation in net W through N‐doping engineering coupled with N position regulation strategies. (b) Crystal structure of net W, where the black dashed lines denote the primitive cell. The four‐, six‐, and eight‐membered rings are highlighted in blue, red, and green, respectively. (c) Band structures of net W calculated using the PBE (blue solid lines) and HSE06 (red dashed lines) functionals. (d) Phonon dispersion relations of net W.

Available strategies for modulating the electronic structure of biphenylene‐based materials include hydrogenation/halogenation [12, 13, 14], nanoribbon construction [2, 15], strain engineering [16], and heteroatom doping [17, 18]. However, their effectiveness has proven to be limited. Notably, although doping engineering (particularly N‐doping) is widely recognized as an effective approach for tuning the electronic structure of 2D carbon materials, current studies on trace N‐doping in biphenylene systems have failed to induce bandgap opening [17, 19, 20]. This limitation can be attributed to the low N‐doping concentration, as concentration‐dependent electronic modulation has been demonstrated in hydrogenated and halogenated biphenylene systems [12, 21]. To address this issue, we theoretically investigate the modulation effects of high‐concentration N‐doping and doping‐site engineering on the electronic structure of net W [7, 22, 23]. Initially, a Dirac semimetal, THO‐C_3_N‐1, is obtained through N‐doping engineering, and its charge distribution, stability, Fermi velocity, and orbital origin of Dirac states are systematically studied using first‐principles calculations. The symmetry‐protected mechanism of the Dirac cone is elucidated, and the nontrivial topological nature of THO‐C_3_N‐1 is confirmed by calculating the Z_2_ invariant and edge‐state. Subsequently, by adjusting the N‐doping sites, two carbon nitride semiconductors, THO‐C_3_N‐2 and THO‐C_3_N‐3, are identified. Their dynamic, thermal, and mechanical stability are comprehensively evaluated. Furthermore, the potential applications of THO‐C_3_N‐2 and THO‐C_3_N‐3 in optics and optoelectronics are explored through calculations of optical absorption spectra and carrier mobility. This work achieves a continuous electronic structure transition in net W from metal to Dirac semimetal and ultimately to semiconductor (Figure 1a). The proposed band engineering strategy provides theoretical guidance for modulating the electronic properties of biphenylene‐based materials.

## 2 Results and Discussion

Here, net W is selected as the prototype material for a detailed investigation of its structural and electronic properties, as well as electronic structure modulation via doping engineering. This selection is motivated by previous studies indicating that net W possesses distinct energetic and symmetry advantages over the biphenylene network, suggesting superior structural stability [2, 7]. Figure 1b illustrates the crystal structure of net W, which belongs to the space group *Cmmm* (No. 65). The rhombic region enclosed by black dashed lines represents the primitive cell. Structural analysis reveals that the primitive cell of net W contains eight carbon atoms, which can be equally divided into two distinct types (C_1_ and C_2_). Specifically, four C_1_ atoms form C_668_ configurations, while the remaining four C_2_ atoms constitute C_468_ configurations. The notations C_668_ and C_468_ denote carbon atoms participating in different ring combinations. The optimized lattice constants for net W are *a*_1_ = *a*_2_ = 5.49 Å with an acute angle *γ* = 47.73°, consistent with previous reports [2, 8]. The band structures of net W along high‐symmetry paths in the first Brillouin zone (see Figure S1) are calculated using both PBE and HSE06 functionals. As illustrated in Figure 1c, both methods confirm the metallic nature of net W, further supported by calculations of the total density of states (TDOS). To evaluate the dynamic stability of net W, its phonon dispersion relations are calculated using the DFPT method. As shown in Figure 1d, the absence of imaginary frequencies along all high‐symmetry paths demonstrates excellent dynamic stability. Despite its excellent structural stability, the intrinsic metallic nature of net W renders it unsuitable for applications in logic devices. Therefore, strategic modulation of its electronic structure becomes imperative for functional applications.

**FIGURE 2:**
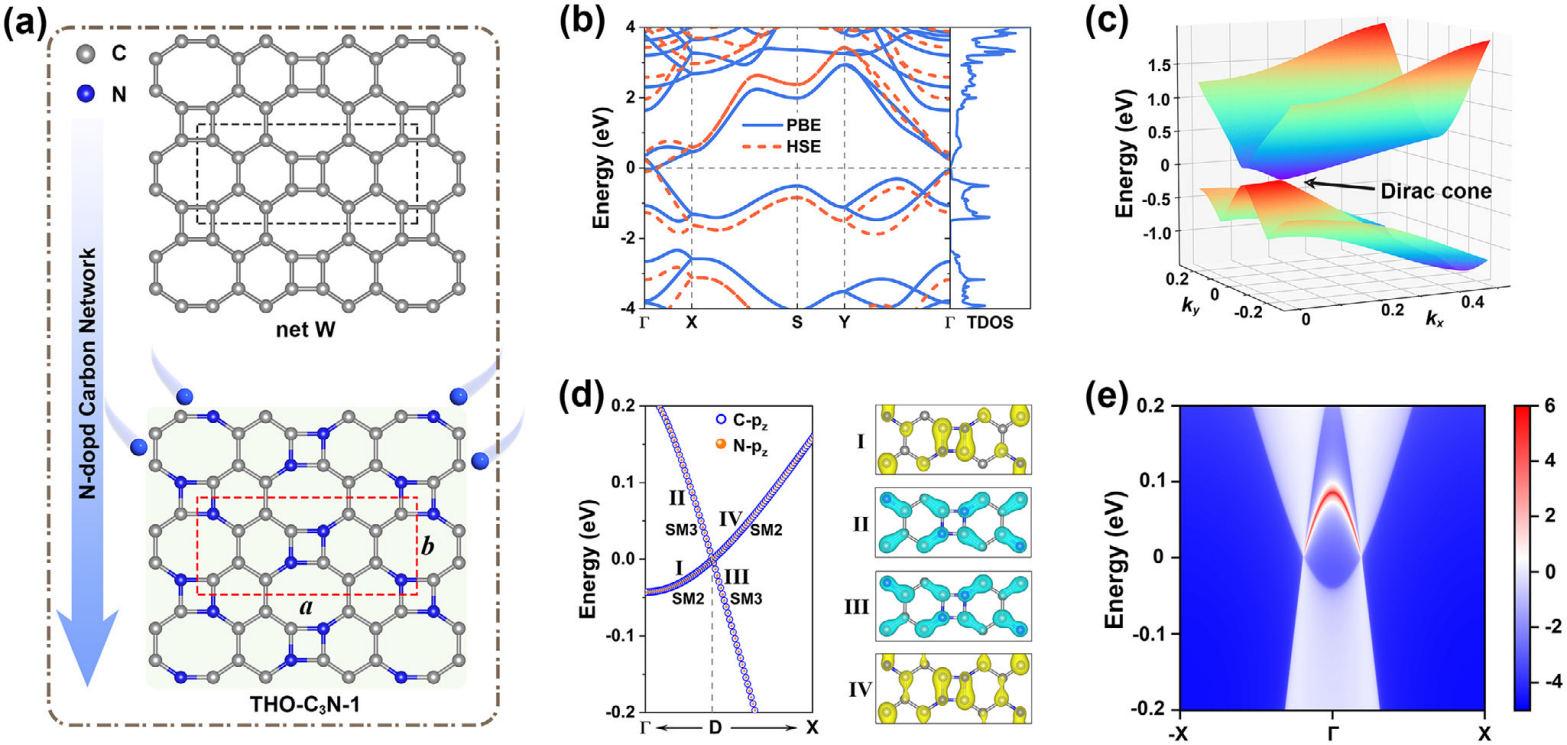
(a) Schematic illustration of the transformation from net W to THO‐C_3_N‐1 via N‐doping engineering. The black and red dashed lines represent the unit cell of net W and THO‐C_3_N‐1, respectively. (b) Band structures of THO‐C_3_N‐1 calculated using the PBE (blue solid lines) and HSE06 (red dashed lines) functionals. (c) 3D band structure in the vicinity of the Dirac cone. (d) Orbital‐resolved band structure and band‐decomposed charge density distributions of THO‐C_3_N‐1 around the Dirac cone. (e) Edge density of states of THO‐C_3_N‐1 with a specific edge termination.

Nitrogen doping has been widely recognized as an effective strategy for modulating the chemical composition and electronic structure of carbon materials [24, 25]. On one hand, nitrogen atoms possess comparable atomic radii and electronegativity to carbon atoms. Each nitrogen atom contributes five valence electrons occupying the 2*s* and 2*p* orbitals, enabling strong bonding with carbon atoms. Specifically, three valence electrons form σ bonds, one electron occupies the π state, while the fifth electron populates the π* state in the conduction band, resulting in significant *n*‐type doping effects. On the other hand, nitrogen doping can modify the symmetry and charge density distribution of carbon networks, thereby tuning their intrinsic properties [26]. However, it should be noted that low doping concentrations often fail to achieve the desired electronic modulation effects, as demonstrated by previous studies showing that single N‐atom substitution cannot open the bandgap in the biphenylene network [19, 20, 27]. To effectively engineer the electronic structure of net W, we implemented a strategy of high‐concentration nitrogen substitutional doping based on its conventional unit cell, which preserves the partial crystal symmetry that is absent in the primitive cell. As illustrated in Figure 2a, by replacing specific carbon atoms on the C_4_‐rings while preserving a certain level of crystal symmetry, a new 2D carbon nitride network is obtained. Given that this structure is composed of tetra‐, hexa‐, and octagonal rings and exhibits a C: N atomic ratio of 3:1, it is denoted as THO‐C_3_N‐1, where the suffix “−1” distinguishes it as the first such C_3_N structure proposed in this work. Symmetry analysis reveals that THO‐C_3_N‐1 belongs to the *Pbam* space group (No. 55), featuring an orthorhombic lattice with 12 carbon atoms and 4 nitrogen atoms per unit cell. The optimized lattice constants are *a* = 9.80 Å and *b* = 4.39 Å. Notably, all nitrogen atoms in THO‐C_3_N‐1 are graphitic N, and electron localization function (ELF) analysis confirms the formation of distinct C–C and C–N covalent bonds (see Figure S2). These findings suggest the existence of σ‐type *sp* ^2^ hybridization throughout the carbon nitride network. Quantitatively, Bader charge analysis reveals that each carbon atom in THO‐C_3_N‐1 donates an average of 0.41 electrons, while each nitrogen atom gains 1.23 electrons. This electron transfer from carbon to nitrogen can be attributed to their electronegativity difference and facilitates the formation of a robust covalent network.

The stability of THO‐C_3_N‐1 is systematically investigated through multiple approaches. Phonon spectrum calculation (see Figure S3) revealed no imaginary frequencies across the entire Brillouin zone, confirming its dynamic stability. Furthermore, AIMD simulations under the NPT ensemble demonstrated excellent thermal stability, with the lattice structure remaining intact after 6 ps at 500 K and even at 1000 K (see Figure S4). The electronic structure of THO‐C_3_N‐1 is then examined using the PBE functional. As shown in Figure 2b, the band structure exhibits a zero bandgap, with two nearly linear bands crossing at the Fermi level. The crossing occurs at an off‐symmetry point (0.103, 0) between Γ and X, and the corresponding charge carriers can be described as massless Dirac fermions. Moreover, the TDOS shows a vanishing value at the Fermi level, excluding band folding as the origin of the Dirac point. HSE06 hybrid functional calculations (red dashed lines, Figure 2b) confirm the persistence of Dirac states in THO‐C_3_N‐1 despite increased band dispersion. As shown in Figure S5, the spin‐orbit coupling (SOC) effect induces only a negligible bandgap (≈0.44 meV), consistent with the weak relativistic effects of light elements (C and N). The 3D band structure (Figure 2c) reveals a distorted Dirac cone, suggesting direction‐dependent Fermi velocities similar to those in 2D ladder polyborane [28] and TPH‐C_5_N_3_ [29]. By linearly fitting the bands near the Dirac point, the Fermi velocity of Dirac fermions is extracted using the expression *v_f_* = ∂*E*/(*ℏ*∂*k*). The direction‐dependent Fermi velocity (see Figure S6) forms an approximate “8” shaped curve, revealing significant anisotropy. The maximum and minimum Fermi velocities are 1.43 × 10^5^ m/s and 0.19 × 10^5^ m/s, respectively. Moreover, the anisotropy of the Dirac cone can be further quantified using the expression A(D) *=* (*v*_max_(*θ*) − *v*_min_(*θ*)) / (*v*_max_(*θ*) + *v*_min_(*θ*)), where *v*_max_(*θ*) and *v*_min_(*θ*) represent the maximum and minimum Fermi velocities over the full 2π range [30]. The calculated A(D) for THO‐C_3_N‐1 is 76.5%, which is comparable to that of PHH‐graphene (71.2%) [31], and significantly higher than those of TPH‐C_5_N_3_ (55.8%) [29] and phagraphene (25.4%) [31].

To investigate the orbital origin of the Dirac states in THO‐C_3_N‐1, we calculated the orbital‐resolved band structure near the Dirac point. As shown in Figure 2d, the Dirac states of THO‐C_3_N‐1 primarily originate from the *p*_z_ orbitals of C and N atoms, with the *p*_z_ orbital of C being dominant. In addition, symmetry analysis of the crossing bands is performed using the IRVSP code [32], revealing that the irreducible representations (IRs) of the two crossing bands are SM2 and SM3, respectively. The bands on either side of the crossing point belong to distinct IRs, indicating that the Dirac cone in THO‐C_3_N‐1 originates from an unavoidable band crossing. Moreover, along the high‐symmetry path Γ‐X, the corresponding point group is *C*_2_ *_v_*, and the Dirac states are protected by the off‐centered screw rotation symmetry $2_{100}(\frac{1}{2},\frac{1}{2},0)$ [33]. To gain further insight, we selected specific points (labeled I‐IV in the left panel of Figure 2d) on the two bands forming the Dirac cone and calculated their band‐decomposed charge density distributions. The results show that positions I and III, as well as II and IV, exhibit similar charge density distributions (right panel of Figure 2d), confirming that the Dirac point in THO‐C_3_N‐1 indeed results from an unavoidable band crossing. Simultaneously, it can be visually confirmed that all four sites exhibit charge density distributions characteristic of *p*_z_ orbital π‐bonding states, which is consistent with the analysis of the orbital‐resolved band structure described above.

Mechanistically, the transition from metallic to Dirac semimetallic band structure induced by N‐doping can be attributed to the electron‐rich nature of N, which introduces an *n*‐type doping effect and leads to an upward shift of the Fermi level. A detailed comparison of the band structures is provided in Figure S7, where the substitution doping of four N atoms into net W results in a Fermi level shift of ≈1.91 eV. This behavior is analogous to that observed in graphene doped with graphitic N [34].

The semimetallic nature of THO‐C_3_N‐1 is further confirmed by investigating its nontrivial topological properties. Due to the presence of both inversion and time‐reversal symmetries in THO‐C_3_N‐1, we evaluated its topological nature by calculating the Z_2_ invariant. Specifically, the Z_2_ invariant can be determined by examining the parity of the occupied Bloch wave functions at the TRIM points in the Brillouin zone [35]. For THO‐C_3_N‐1, the four TRIM points are Γ, X, S, and Y. The parity eigenvalues of the occupied bands at each TRIM point are obtained using the IRVSP code [32]. Furthermore, the product of the parity eigenvalues (*δ_i_*) of the occupied bands at each TRIM point is calculated using Equation (1), and the results are summarized in Table 1. Subsequently, the Z_2_ invariant *ν* is obtained using Equation (2), yielding a value of *ν* = 1. This result clearly indicates the existence of a nontrivial topological semimetal state in THO‐C_3_N‐1.

**TABLE 1:** Parity products *δ_i_* of the occupied bands at TRIM points and the corresponding Z_2_ invariant for the THO‐C_3_N‐1 monolayer.

Z_2_ invariant *v*	*δ*_Γ_	*δ*_X_	*δ*_S_	*δ*_Y_
1	−1	−1	+1	−1

The nontrivial topological nature of THO‐C_3_N‐1 is further characterized by the presence of topologically protected edge states, a hallmark of 2D topological materials that arises from the bulk‐boundary correspondence principle. To this end, we employed a tight‐binding Hamiltonian constructed with Wannier90, combined with the iterative Green's function method to calculate the edge states of semi‐infinite THO‐C_3_N‐1 [36, 37]. As shown in Figure 2e, a clear topological edge state is observed, connecting two adjacent Dirac points, which provides compelling evidence for the nontrivial topological semimetallic nature in THO‐C_3_N‐1. These results demonstrate that the N‐doping strategy effectively modulates net W from a metal to a Dirac semimetal. However, the gapless nature still limits its potential applications in nanoelectronics. Therefore, further optimization is required to open a band gap.

**FIGURE 3:**
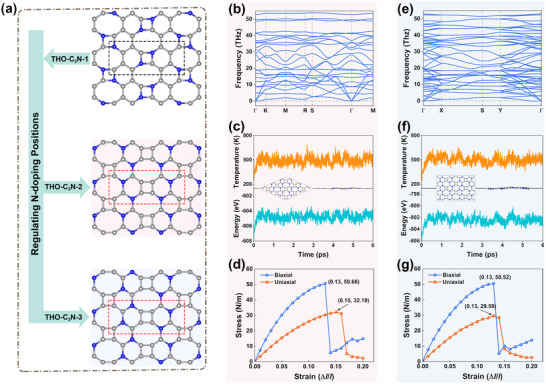
(a) Schematic illustration of the structural evolution from THO‐C_3_N‐1 to THO‐C_3_N‐2 and THO‐C_3_N‐3 via regulation of N‐doping positions. Phonon spectra of (b) THO‐C_3_N‐2 and (e) THO‐C_3_N‐3. Temperature and total energy variations during AIMD simulations under NPT ensemble at 500K for (c) THO‐C_3_N‐2 and (f) THO‐C_3_N‐3. The inset in each panel shows the final atomic structure after the AIMD simulation. Uniaxial and biaxial stress‐strain curves for (d) THO‐C_3_N‐2 and (g) THO‐C_3_N‐3.

Previous studies have demonstrated that tuning the doping sites of atoms can effectively modulate the electronic structure [27, 38]. Meanwhile, experimental methodologies for site‐specific N‐doping are now highly developed. For instance, site‐specific N‐doping has been successfully implemented in graphdiyne [39], quasi‐1D covalent organic frameworks [40], and carbon fibers [41]. Herein, by adjusting the N doping sites, we achieved effective modulation of the electronic structure starting from THO‐C_3_N‐1. Specifically, as illustrated in Figure 3a, by relocating the N atoms to the C_668_ sites, two new carbon nitride monolayers, THO‐C_3_N‐2 and THO‐C_3_N‐3, are obtained, differing in the absence or presence of inversion symmetry. Crystallographic analysis reveals that THO‐C_3_N‐2 and THO‐C_3_N‐3 belong to the *Amm*2 (No. 38) and *Pbam* (No. 55) space groups, respectively, both featuring orthorhombic lattices. After structural optimization, the lattice constants are determined to be *a*_1_ = *a*_2_ = 5.49 Å for THO‐C_3_N‐2, and *a* = 10.04 Å, *b* = 4.44 Å for THO‐C_3_N‐3. The average C─C bond lengths in THO‐C_3_N‐2 and THO‐C_3_N‐3 are 1.41 and 1.42 Å, respectively, comparable to that in graphene (1.42 Å). Meanwhile, the C─N bond lengths are 1.39 and 1.38 Å, respectively, close to those in the synthesized 2D C_3_N crystalline (1.40 Å) [42]. More detailed lattice parameters of the two structures are summarized in Table S1.

The dynamical stability of THO‐C_3_N‐2 and THO‐C_3_N‐3 is evaluated by calculating their phonon dispersion relations using the DFPT method. The first Brillouin zones and high‐symmetry paths for both monolayers are shown in Figure S8. The calculated phonon spectra (Figure 3b,e) reveal no imaginary frequencies along any high‐symmetry paths, demonstrating excellent dynamical stability for both structures. Moreover, the high frequencies of the optical phonon branches provide further evidence for the structural stability of both monolayers [43]. Specifically, the highest phonon modes reach 53.84 and 52.91 THz for THO‐C_3_N‐2 and THO‐C_3_N‐3, respectively, which are higher than those of synthesized biphenylene (49.71 THz) [44], C_3_N (49.49 THz) [45], and C_2_N (46.98 THz) [43], indicating the formation of strong covalent bonds in both THO‐C_3_N monolayers. Furthermore, AIMD simulations under the NPT ensemble are conducted to assess their thermal stability. As illustrated in Figure 3c,f, the temperature and energy fluctuations at 500 K remain within a narrow range over time, and the atomic configurations retain their planar structures after 6 ps of simulation. These results confirm the excellent thermal stability of both monolayers. Additional AIMD simulations at higher temperature (1000 K) reveal that both monolayers maintain their structural integrity without bond breaking or reconstruction (see Figure S9), highlighting their exceptional thermal stability and confirming their suitability for high‐temperature synthesis.

The mechanical stability of THO‐C_3_N‐2 and THO‐C_3_N‐3 monolayers is examined by calculating their linear elastic constants using the energy‐strain method. For THO‐C_3_N‐2, mechanical properties are evaluated using the conventional unit cell to facilitate strain application. The calculated elastic constants are summarized in Table S2. Both sets of elastic constants satisfy the Born stability criteria [46], i.e., *C*_11_ *C*_22_ − *C*_12_^2^> 0 and *C*_66_> 0, confirming the mechanical stability of both monolayers. Furthermore, based on Equations ((3), (4)), we plotted the angle‐dependent Young's modulus *E*(*θ*) and Poisson's ratio *ν*(*θ*) to investigate their stiffness and mechanical response to external loading. As shown in Figure S10, both monolayers exhibit anisotropic characteristics in *E*(*θ*) and *ν*(*θ*), originating from the inherent orthorhombic symmetry of the two structures. Although the Young's moduli of THO‐C_3_N‐2 and THO‐C_3_N‐3 monolayers are lower than those of graphene (348 N/m) and synthesized C_3_N (356 N/m) [47], they exceed those of biphenylene (212.4–259.7 N/m) [44], indicating that THO‐C_3_N‐2 and THO‐C_3_N‐3 combine rigidity with flexibility.

The ideal strength, a crucial intrinsic mechanical property representing the maximum stress a material can withstand before bond breaking, is investigated for THO‐C_3_N‐2 and THO‐C_3_N‐3 by calculating uniaxial (along the *x* direction) and biaxial stress‐strain curves. As shown in Figure 3d,g, within the harmonic region, the stress exhibits an approximately linear response to increasing strain, corresponding to linear elastic behavior. Upon further strain application, the stress increases nonlinearly until structural failure occurs. Notably, under identical strain conditions, the stress induced by biaxial strain in both monolayers is approximately twice that generated by uniaxial strain. The calculated ultimate strengths along the *x* direction for THO‐C_3_N‐2 and THO‐C_3_N‐3 are 32.19 N/m and 29.59 N/m, respectively, with corresponding ultimate tensile strains of 15% and 13%. Remarkably, while these ultimate strengths exceed that of biphenylene (28.81 N/m), the ultimate strains are lower than that of biphenylene (22%) [44], indicating greater rigidity but reduced ductility. Furthermore, their ideal strengths significantly surpass those of synthesized 2D C_3_N_4_ (9.5 N/m, 11%) and C_3_N_5_ (3.2 N/m, 12%) [48], suggesting promising potential for applications in nanomechanical devices.

To further investigate the energetic stability of the THO‑C_3_N monolayers, crystal structure prediction is performed by combining a random search strategy with group theory and graph theory [49], guided by key structural search parameters. These parameters included the space group range (No. 19‒64), the number of inequivalent atoms for C and N (≤ 3), the total number of atoms per unit cell (≤ 20), coordination numbers, bond lengths and angles, and the ring sequence (required to be composed of four‐, six‐, and eight‐membered rings). After excluding chemically unreasonable structures, we identified 21 new 2D carbon nitride monolayers built from 4‐6‐8 ring motifs, which include the three THO‐C_3_N monolayers. As shown in Figure S11, these 2D carbon nitride structures can be categorized into five stoichiometries with C:N ratios of 4:1, 3:1, 2:1, 3:2, and 1:1. To quantify the relative stability of these 2D carbon nitrides and evaluate their synthetic potential, their cohesive energies are calculated using the Equation (5). As shown in Figure S12, by calculating the cohesive energies of the 21 monolayers and constructing the convex hull, we found that the most negative (most stable) structure is THO‐C_3_N‐1 (−6.78 eV/atom). Notably, although THO‐C_3_N‐2 (−6.66 eV/atom) and THO‐C_3_N‐3 (−6.63 eV/atom) appear as metastable structures and are not located on the convex hull stability line, their energies are still lower than those of the synthesized C_3_N_3_ (−6.24 eV/atom), C_3_N_4_ (−6.05 eV/atom), and C_3_N_5_ (−5.81 eV/atom) [50]. Since a more negative cohesive energy indicates greater energetic stability, from the perspective of cohesive energy, all three THO‐C_3_N monolayers exhibit energetic feasibility for experimental synthesis. Furthermore, the synthesizability of the three THO‐C_3_N monolayers is further assessed using the formation energy calculated by Equation (6). For comparison, we also calculated the formation energies of experimentally synthesized 2D C_2_N, C_3_N, C_3_N_3_, C_3_N_4_, C_3_N_5_, and C_4_N_3_ materials, and the results are consistent with previously reported values [51, 52, 53]. It should be noted that, despite being successfully synthesized and widely applied, all these 2D carbon nitride materials exhibit positive formation energies. As shown in Figure S13, the three proposed THO‐C_3_N monolayers exhibit formation energies of 0.138 eV/atom, 0.251 eV/atom, and 0.285 eV/atom, respectively. These values are comparable to those of synthesized C_2_N, C_3_N, C_3_N_4_, and C_3_N_3_, and significantly lower than those of synthesized C_3_N_5_ and C_4_N_3_, indicating that all three monolayers possess relative energetic stability and potential for experimental synthesis.

**FIGURE 4:**
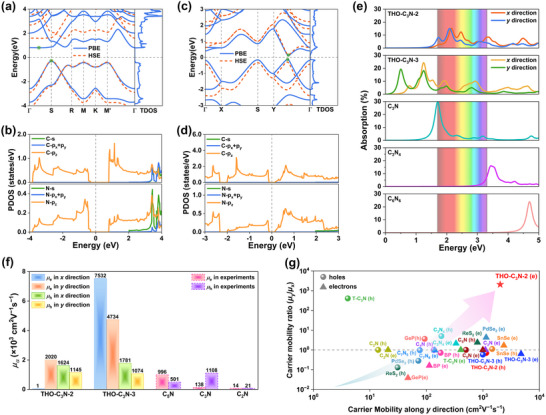
Band structures of (a) THO‐C_3_N‐2 and (c) THO‐C_3_N‐3 calculated using the PBE (blue solid lines) and HSE06 (red dashed lines) functionals. Projected density of states for (b) THO‐C_3_N‐2 and (d) THO‐C_3_N‐3. (e) Calculated absorbances of THO‐C_3_N‐2 and THO‐C_3_N‐3 in comparison with synthesized 2D carbon nitrides at the G_0_W_0_ + BSE level. (f) Comparison of calculated carrier mobility of THO‐C_3_N‐2 and THO‐C_3_N‐3 with experimental carrier mobility of C_2_N, C_3_N, and C_5_N. (g) Comparison of carrier mobility ratios for THO‐C_3_N‐2 and THO‐C_3_N‐3 with those of 2D carbon nitrides and typical anisotropic 2D materials.

Having confirmed the stability of THO‐C_3_N‐2 and THO‐C_3_N‐3, we investigated their electronic structures. The band structures and TDOS for both monolayers are first calculated using the PBE functional. As shown in Figure 4a,c, THO‐C_3_N‐2 and THO‐C_3_N‐3 exhibit indirect semiconductor characteristics. Specifically, the calculated bandgaps are 1.11 and 0.23 eV for THO‐C_3_N‐2 and THO‐C_3_N‐3, respectively, with the valence band maximum (VBM) and conduction band minimum (CBM) indicated by green dots in Figure 4a,c. HSE06 calculations (red dashed lines) yield larger bandgaps of 1.84 and 0.46 eV for THO‐C_3_N‐2 and THO‐C_3_N‐3, respectively. The bandgap of THO‐C_3_N‐2 is comparable to that of synthesized C_3_N_5_ (1.76 eV) [54], while THO‐C_3_N‐3 exhibits a bandgap similar to the theoretically proposed T‐C_3_N_2_ (0.35 eV) [50]. In addition, the origin of the electronic properties is further investigated by calculating the projected density of states (PDOS). As shown in Figure 4b,d, both the VBM and CBM of THO‐C_3_N‐2 and THO‐C_3_N‐3 primarily arise from the out‐of‐plane *p*_z_ orbitals of C and N atoms. The local 3D band structures (see Figure S14) further confirm the semiconducting nature of both monolayers. These results collectively demonstrate that the transition from Dirac semimetal to intrinsic semiconductor can be achieved by strategically tuning the doping positions of N atoms.

To elucidate the origin of the transition from Dirac semimetallic to semiconducting behavior, detailed comparative analyses of the band structures of the three THO‐C_3_N monolayers are conducted. As shown in Figure S15a, the electronic structures of the three THO‐C_3_N monolayers are primarily determined by the dispersion of four bands near the Fermi level. When the N‐doping site shifts from C_468_ to C_668_, the curvature of the two bands forming the Dirac cone in THO‐C_3_N‐1 is altered. Specifically, reduced band dispersion along the Γ–X path eliminates the band crossing and opens a bandgap. To gain deeper insight, band‐decomposed charge density analyses are performed at the Dirac point (0.103, 0, 0) for the lowest conduction band (LCB) and highest valence band (HVB) of all three structures. Figure S15b–g reveals a significant redistribution of charge density in THO‐C_3_N‐2 and THO‐C_3_N‐3 compared to THO‐C_3_N‐1. For instance, THO‐C_3_N‐2 exhibits reduced charge density at C–N bonds in the HVB, while THO‐C_3_N‐3 shows enhanced charge density at C─C bonds in the LCB. Furthermore, ELF analysis (Figure S16a–c) demonstrates that modifying the N‐doping sites induces subtle shifts in electron localization centers of C─C and C─N bonds. Deformation charge density analysis further indicates that adjustment of the N‐doping sites induces more pronounced charge depletion in six‐membered rings while minimally affecting four‐ and eight‐membered rings Figure S16d–f). Quantitatively, Bader charge analysis reveals that compared with THO‐C_3_N‐1, the charge transfer between C and N atoms decreases in THO‐C_3_N‐2 but increases in THO‐C_3_N‐3 (see Table S3). As a result, the transition from Dirac semimetal to semiconductor can be attributed to modifications in the local charge distribution induced by the change in N‐doping sites, which shift the band edge positions near the Fermi level and ultimately open the band gap. This doping‐site‐dependent evolution of electronic structures has also been reported in systems such as B/N‐substituted phagraphene nanoribbons [55] and N‐doped biphenylene [27].

Overall, the metal‐Dirac‐semiconductor transition induced by N‑doping in net W exemplifies a prototypical point (impurity) defect. This type of impurity defect does not alter the fundamental lattice of the net W matrix but induces significant structural distortions [56]. Specifically, N‑doping introduces lone‑pair electrons, modifies the surface polarity of net W through copolymerization with C, and the higher electronegativity of N promotes electron redistribution, thereby profoundly influencing the electronic structure of net W. More fundamentally, introducing point defects into the parent net W leads to various configurations, namely THO‐C_3_N‐1, THO‐C_3_N‐2, and THO‐C_3_N‐3. The evolution of their band structures is governed by four mutually coupled factors: (i) Structural distortion: Different doping configurations introduce varying degrees of structural distortion, leading to subtle yet critical modifications in the dispersion relations of individual bands. (ii) Band‐filling (electron‐count) effect: In each unit cell of the THO‐C_3_N monolayers, four C atoms are substituted by N atoms. Since each N atom contributes one more valence electron than C, this substitution introduces four extra electrons per unit cell. In the undoped parent lattice, the Fermi level lies between the *n*‐th and (*n* + 1)‐th bands. After N substitution, the extra electrons must be accommodated by the band structure, shifting the effective occupation to higher bands and moving the Fermi level to lie between the (*n* + 2)‐th and (*n* + 3)‐th bands. (iii) On‐site energy differences: As N atoms occupy distinct lattice sites, their local on‐site energies vary. Consequently, bands with dominant contributions from N atoms undergo relative shifts, reconstructing the band structure near the Fermi level that governs low‐energy physics. (iv) Symmetry and band folding: Due to the formation of supercells and the resulting band folding, two sets of band crossings, *n*/(*n* + 1) and (*n* + 2)/(*n* + 3), emerge along high‐symmetry paths such as Γ‐X and Γ‐Y. Whether these crossings are preserved or gapped depends on the crystalline symmetry corresponding to the specific N‐doping pattern. For example, the Dirac crossing in THO‐C_3_N‐1 is protected by off‐centered screw rotation symmetry. The results from band‑decomposed charge density, ELF, deformation charge density, and Bader charge analyses consistently reflect the electronic redistribution that accompanies this band reconstruction, thereby corroborating the above scenario.

The semiconductor characteristics of THO‐C_3_N‐2 and THO‐C_3_N‐3 suggest potential applications in photovoltaics and optoelectronics. The energy‐dependent optical absorption spectra of the two monolayers are calculated within the G_0_W_0_ + BSE approach. As shown in Figure 4e, the optical absorption spectra reveal distinct anisotropies between the *x* and *y* directions for both monolayers. Additionally, both exhibit obvious visible‐light absorption capabilities. Specifically, the peak visible‐light absorbances along the *x*(*y*) direction reach 15.3% (15.1%) for THO‐C_3_N‐2 and 10.3% (6.3%) for THO‐C_3_N‐3, which are slightly lower than those of synthesized C_3_N but significantly higher than those of the C_3_N_4_ and C_6_N_6_. Notably, owing to its narrow bandgap characteristic, THO‐C_3_N‐3 exhibits an additional prominent absorption peak in the NIR region, with absorbances of 24.4% and 18.2% along the *x* and *y* directions, respectively. Considering that solar radiation reaching the Earth's surface primarily spans the visible and NIR regions, THO‐C_3_N‐2 and THO‐C_3_N‐3 emerge as promising candidates for photonic and optoelectronic applications.

To characterize the carrier transport properties of THO‐C_3_N monolayers, we investigated the carrier mobility under various scattering mechanisms by the AMSET package [57]. This computational framework incorporates multiple important scattering mechanisms: acoustic deformation potential scattering (ADP), polar optical phonon scattering (POP), and ionized impurity scattering (IMP), and integrates them via Matthiessen's rule Equation (7) to provide a comprehensive assessment of carrier mobility. Due to the orthorhombic lattice of both monolayers, conventional unit cells are employed for mobility calculations, with the zigzag and armchair directions defined as the *x* and *y* axes, respectively (see Figure S17). As illustrated in Figures S18 and S19, we calculated the temperature‐dependent carrier mobility for the THO‐C_3_N‐2 and THO‐C_3_N‐3 at a carrier concentration of 1 × 10^16^ cm^−3^. Notably, this concentration aligns with experimental values commonly reported for mainstream synthesized 2D materials [54, 58, 59]. The results indicate that the total carrier mobility exhibits a negative correlation with temperature. According to Matthiessen's rule, as multiple carrier scattering mechanisms couple, the total mobility of THO‐C_3_N semiconductors decreases significantly, approaching the value of the mechanism with the lowest individual mobility. Specifically, ADP scattering plays a dominant role. With increasing temperature, the influence of POP scattering becomes increasingly important due to the rising population of optical phonons under higher thermal energy, which enhances electron‒phonon interactions. Additionally, IMP scattering exhibits a notable influence at low temperatures, as the reduced average carrier velocity makes it more difficult for carriers to bypass charged impurities.

Moreover, as shown in Figure 4f, the results reveal significant differences in carrier properties along the *x* and *y* directions for both THO‐C_3_N‐2 and THO‐C_3_N‐3, which result in anisotropic electron (hole) mobilities. Specifically, at room temperature, the electron mobility ratios for THO‐C_3_N‐2 and THO‐C_3_N‐3 are found to be 2061.22 and 0.63, respectively, while the hole mobility ratios are 0.71 and 0.60. This anisotropy originates from the intrinsic orthorhombic symmetry of both structures. Notably, THO‐C_3_N‐2 exhibits an exceptionally high electron mobility of 2020.46 cm^2^ V^−1^ s^−1^ along the *y* direction, while THO‐C_3_N‐3 achieves a remarkable electron mobility of 7531.53 cm^2^ V^−1^ s^−1^ along the *x* direction. Additionally, both THO‐C_3_N‐2 and THO‐C_3_N‐3 demonstrate hole mobilities exceeding 10^3^ cm^2^ V^−1^ s^−1^ along both the *x* and *y* directions, far exceeding those of synthesized C_2_N [60], C_3_N [61], and C_5_N [64].

It is particularly noteworthy that the exceptionally high electron mobility ratio of THO‐C_3_N‐2 surpasses those of extensively studied 2D carbon nitrides, including C_2_N [60], C_3_N [61], T‐C_3_N [62], C_3_N_4_ [63]_,_ C_5_N [64], and C_7_N_6_ [65], while also significantly exceeding those of typical anisotropic 2D materials such as black phosphorus [66], SnSe [67], GeP [68], PdSe_2_ [69], and ReS_2_ [70]. The carrier mobility ratios of these related structures are comparatively illustrated in Figure 4g. The anisotropic charge transport properties and high carrier mobilities of THO‐C_3_N‐2 and THO‐C_3_N‐3 make them highly suitable for advanced flexible electronics and optoelectronic applications.

Given the dynamic, thermal, and mechanical stability of THO‐C_3_N‐2 and THO‐C_3_N‐3 monolayers, along with their outstanding optoelectronic properties, their feasible experimental synthesis routes warrant attention. Drawing from reported cases of 2D carbon nitrides synthesis, the selection of appropriate precursors and compatible experimental approaches is crucial for successful fabrication. For instance, honeycomb nonporous C_3_N can be synthesized through direct pyrolysis of hexaaminobenzene trihydrochloride (HAB) or polymerization of 2,3‐diaminophenazine (DAP) [71, 72]. Similarly, g‐C_3_N_4_ can be obtained via direct thermal decomposition of melamine under vacuum [73]. Moreover, optimizing experimental conditions, including monomer concentration, temperature, pressure, and reaction duration, is equally critical. For example, crystalline C_3_N formation requires temperatures ≥ 510 K, as lower temperatures yield amorphous polymers [72], while g‐C_3_N_4_ synthesis necessitates medium temperatures to preserve triazine rings during melamine pyrolysis [73]. Herein, we rationally designed synthesis routes for THO‐C_3_N‐2 and THO‐C_3_N‐3 (see Figure S20) by analyzing their structural features alongside current progress in 2D carbon nitrides synthesis: (i) For THO‐C_3_N‐2, the 1,2‐dimethylhexahydropyrimidine (C_6_H_14_N_2_) is selected as the precursor. Dehydrogenation cleaves C─H and N─H bonds to generate reactive sites with C/N dangling bonds. (ii) Directionally controlled polymerization forms C─C/C─N bonds. (iii) After releasing excess hydrogen, the target crystalline structure can be obtained. THO‐C_3_N‐3 synthesis follows a similar protocol but involves vertical monomer flipping for molecular reorientation. The process employs a customized hydrothermal system comprising Teflon‐lined stainless‐steel autoclaves and polyphenylene (PPL)‐lined high‐pressure vessels, a setup previously validated for C_3_N synthesis [72]. Specifically, precursors are placed in 100 mL Teflon reactors for ≥ 48 h polymerization under controlled temperatures, yielding ≈100 mg product per batch after centrifugation. To enhance purity, unreacted species and byproducts are removed via semipermeable membrane dialysis.

Finally, to validate the generalizability of the proposed band engineering paradigm, we selected the experimentally synthesized biphenylene network (BPN) for further investigation. As shown in Figure S21a, the pristine BPN unit cell contains six C atoms and exhibits a gapless metallic electronic structure. Subsequently, single‐atom N doping is applied to this structure. The band structure of the resulting C_5_N (Figure S21b) shows that it remains metallic, indicating that low N‐doping concentration is insufficient to significantly modulate its electronic properties. Building on this, we increased the doping level to two N atoms and constructed two distinct configurations, C_2_N‐1 and C_2_N‐2, by adjusting the doping sites. As illustrated in Figure S21c, C_2_N‐1 displays clear Dirac semimetal features, while C_2_N‐2 (Figure S21d) opens a bandgap near the Fermi level, exhibiting semiconductor characteristics. These results demonstrate that by tuning both the N‐doping concentration and the doping sites, a continuous electronic structure transition from metal to semimetal and ultimately to semiconductor can be achieved in the BPN monolayer. This trend aligns with the band engineering mechanism proposed in our work, thereby confirming the generalizability of the strategy.

## 3 Conclusion

In summary, we have developed a band engineering strategy for the biphenylene‐based structure net W. Our results demonstrate that modifying the crystal structure of net W through N‐doping engineering and N sites regulation can significantly alter its electronic properties. Initially, N substitution doping transformed metallic net W into a Dirac semimetal (THO‐C_3_N‐1). Further adjustment of N doping sites achieved a transition from a Dirac semimetal to an intrinsic semiconductor. This secondary transition in the electronic structure is governed by the synergistic interplay of structural distortion, band‐filling effect, on‐site energy differences, and symmetry. The resulting two indirect bandgap semiconductors, THO‐C_3_N‐2 and THO‐C_3_N‐3, exhibit excellent dynamic, thermal, and mechanical stability. Moreover, both semiconductors demonstrate outstanding visible light absorption capabilities. Remarkably, THO‐C_3_N‐2 and THO‐C_3_N‐3 possess high electron mobilities of 2020.46 cm^2^ V^−1^ s^−1^ and 7531.53 cm^2^ V^−1^ s^−1^, respectively. Most unexpectedly, THO‐C_3_N‐2 with a record high electron mobility ratio of 2061.22, a level of anisotropy that far surpasses both 2D carbon nitrides and typical anisotropic 2D materials. These findings not only provide theoretical insights for electronic structure modulation in biphenylene‐based materials, but also expand the potential applications of four‐, six‐, and eight‐membered ring structures in next‐generation optoelectronic devices.

## 4 Computational Methods

All structural optimizations and electronic property calculations are performed using density functional theory (DFT) as implemented in the Vienna ab initio simulation package (VASP) [74]. The projector‐augmented wave (PAW) method is employed to describe electron‐ion interactions [75], while the generalized gradient approximation (GGA) with the Perdew‐Burke‐Ernzerhof (PBE) functional is adopted for exchange‐correlation interactions [76]. A vacuum layer of 15 Å is added along the *z* direction to eliminate interlayer interactions between adjacent periodic images of net W and THO‐C_3_N monolayers. The plane‐wave cutoff energy is set to 500 eV. For structural optimization and self‐consistent calculations, Γ‐centered Monkhorst‐Pack *k*‐point grids of 3 × 9 × 1 and 9 × 9 × 1 are used for primitive and conventional unit cells, respectively. Full structural relaxation is performed until the residual force on each atom is less than 0.001 eV/Å, and the energy convergence threshold is set to 10^−8^ eV. Phonon dispersion relations are calculated using the Phonopy package combined with density functional perturbation theory (DFPT). Ab initio molecular dynamics (AIMD) simulations are conducted in the isothermal‐isobaric ensemble (NPT) at temperatures of 500 K and 1000 K for 6 ps with a time step of 1 fs. Maximally localized Wannier functions are generated using the Wannier90 package [36], and the iterative Green's function method implemented in the WannierTools package [37] is used to determine the edge states of a semi‐infinite structure.

The Z_2_ invariant is determined by evaluating the parity of occupied Bloch wavefunctions at time‐reversal invariant momentum (TRIM) points in the Brillouin zone [35]. Specifically, the product of parity eigenvalues *δ_i_* at each TRIM point is calculated as:

(1) $$\delta_{i}=\prod_{m=1}^{N}\xi_{2m}\left(i\right)$$

where *i* belongs to TRIM point Γ, X, S, and Y. The *ξ*_2m_(*i*) = ± 1 represents the parity eigenvalue of the 2*m*th occupied band at point *i*, which shares the same value with its Kramers degenerate partner *ξ*_2m−1_(*i*). The product runs over all 2N occupied bands. The Z_2_ invariant is then obtained through the product of four *δ_i_* values [35]:

(2) $${\left(-1\right)}^{\nu }=\prod_{i}\delta_{i}$$

Direction‐dependent Young's modulus *E*(*θ*) and Poisson's ratio *ν*(*θ*) are calculated using the following equations [77]:

(3) $$E\left(\theta \right)=\frac{C_{11}C_{22}-C_{12}^{2}}{C_{11}s^{4}+C_{22}c^{4}+\left(\frac{C_{11}C_{22}-C_{12}^{2}}{C_{66}}-2C_{12}\right)s^{2}c^{2}}$$

(4) $$\nu \left(\theta \right)=\frac{C_{12}\left(s^{4}+c^{4}\right)-\left(C_{11}+C_{22}-\frac{C_{11}C_{22}-C_{12}^{2}}{C_{66}}\right)s^{2}c^{2}}{C_{11}s^{4}+C_{22}c^{4}+\left(\frac{C_{11}C_{22}-C_{12}^{2}}{C_{66}}-2C_{12}\right)s^{2}c^{2}}$$

where *s* = sin *θ*, *c* = cos *θ*, and *θ* ranges from 0 to 2π.

To quantify the relative stability of the THO‐C_3_N monolayers and evaluate their synthetic potential, the cohesive energies and formation energies of these structures are calculated using the following equations:

(5) $$E_{coh}=\frac{E_{tot}\left(C_{m}N_{n}\right)-mE_{C}-nE_{N}}{m+n}$$

(6) $$E_{f}=\frac{E_{tot}\left(C_{m}N_{n}\right)-m\mu_{C}-n\mu_{N}}{m+n}$$

where *E*_tot_(C*_m_* N*_n_*), *E*_C_, and *E*_N_ denote the total energies of the C*_m_* N*_n_* system, the energy of an isolated C atom, and the energy of an isolated N atom, respectively. *µ*_C_ and *µ_N_* are the chemical potentials of C and N. The symbols *m* and *n* denote the number of C and N atoms in the 2D C*_m_* N*_n_* monolayer.

To incorporate the effects of many‐body interactions, quasiparticle energies are computed via single‐shot GW (G_0_W_0_) calculations based on DFT results. In the G_0_W_0_ calculations, the energy cutoff is set to 450 eV. A total of 320 bands is used for both THO‐C_3_N‐2 and THO‐C_3_N‐3 to ensure an adequate number of empty bands for excited‐state calculations. The optical absorbance is obtained by solving the Bethe‐Salpeter equation (BSE) with the inclusion of excitonic electron‐hole interactions.

The AMSET package is employed to incorporate acoustic deformation potential scattering (ADP), polar optical phonon scattering (POP), and ionized impurity scattering (IMP) to evaluate the carrier mobility of the proposed THO‐C_3_N semiconductors [57]. The total carrier mobility is calculated by integrating these scattering mechanisms through Matthiessen's rule [78]:

(7) $$\frac{1}{\mu_{\mathrm{total}}}=\frac{1}{\mu_{\mathrm{ADP}}}+\frac{1}{\mu_{\mathrm{IMP}}}+\frac{1}{\mu_{\mathrm{POP}}}$$

where *µ_A_*_DP_, *µ*_IMP_, and *µ*_POP_ represent the mobility contributions limited by ADP, IMP, POP scattering, respectively.

To evaluate the influence of supercell size on the phonon dispersion and POP frequencies, we calculated the phonon spectra of THO‑C_3_N semiconductors using DFPT for different supercell sizes. As shown in Figures S22 and S23, no imaginary frequencies are observed in the phonon spectra of THO‑C_3_N‑2 and THO‑C_3_N‑3 when the supercell size reaches 2 × 3 × 1 or larger, confirming the dynamical stability of both structures. Furthermore, the POP frequencies essentially converge at a supercell size of 2 × 2 × 1, with further enlargement having a negligible effect. These findings demonstrate that DFPT phonon calculations at supercell sizes of 2 × 3 × 1 and above can reliably supply the polar phonon frequencies and dielectric constants needed when incorporating the POP scattering mechanism.

## Conflicts of Interest

The authors declare no conflict of interest.

## Supporting information




**Supporting File**: advs73846‐sup‐0001‐SuppMat.docx.

## Data Availability

The data that support the findings of this study are available from the corresponding author upon reasonable request.
