# Supplementary material for: Semiconducting THO‐C3N Monolayers for Ultrahigh Anisotropic Carrier Mobility
Source: Adv Sci (Weinh). 2026 Jan 15;13(17):e19861. doi: 10.1002/advs.202519861 (PMC13042467; doi:10.1002/advs.202519861)
Supplement: Supplementary file 1 — Supporting File: advs73846‐sup‐0001‐SuppMat.docx. [file ADVS-13-e19861-s001.docx]

**Supporting Information**

**Semiconducting THO-C_3_N Monolayers for ultrahigh Anisotropic Carrier Mobility**

Rui Tan,^1,†^ Xueqing Chen,^1,†^ Jifeng Luo,^2,†^ Zhe Xue,^3,*^ Zehou Li,^1^ Xiaolin Wei,^1^ Zhenkun Tang,^1,*^ and Gaokuo Zhong^2,*^

^1^ *The Key Laboratory of Micro-nano Energy Materials and Application Technologies, University of Hunan Province, College of Physics and Electronic Engineering, Hengyang Normal University, Hengyang 421002, China*

^2^ *Changsha Semiconductor Technology and Application Innovation Research Institute, College of Semiconductors (College of Integrated Circuits), Hunan University, Changsha, 410082 China*

^3^ *School of Materials Science and Engineering, Collaborative Innovation Center of Ministry of Education and Shanxi Province for High-performance Al/Mg Alloy Materials, North University of China, Taiyuan 030051, P R China*

† These authors contributed equally to this work.

* Corresponding authors: gkzhong@hnu.edu.cn, zktang@hynu.edu.cn, xuezhe@nuc.edu.cn.


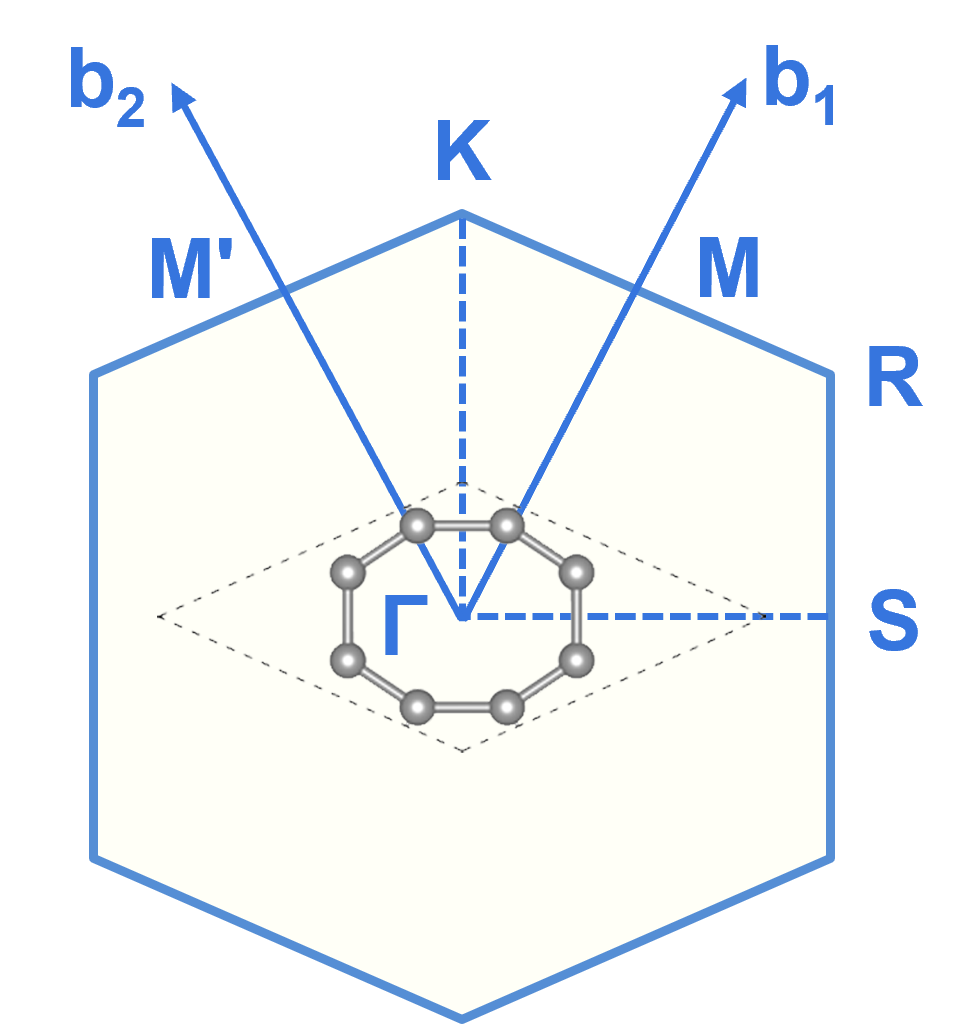


Fig. S1 The first Brillouin zone of net W.


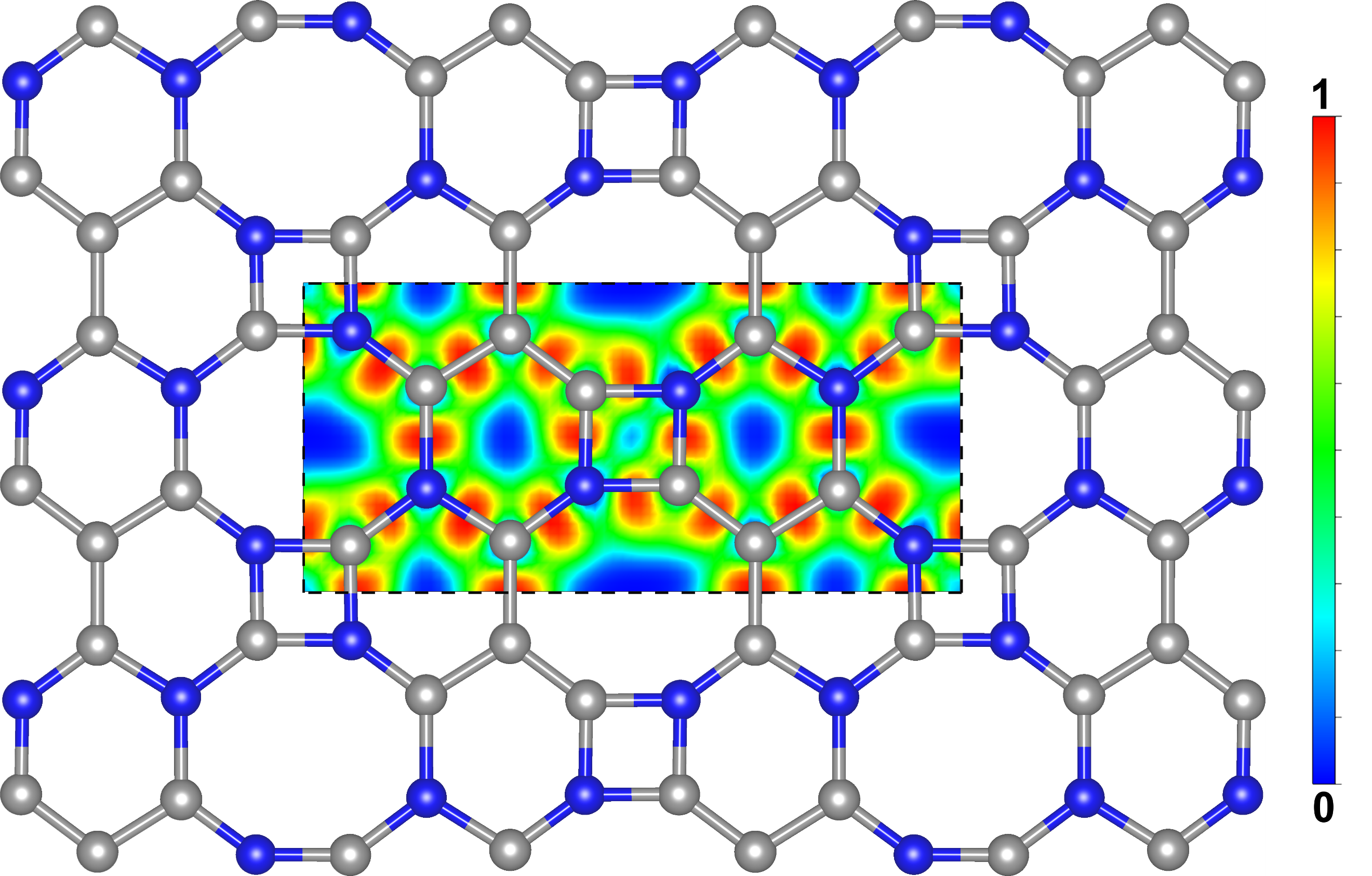


Fig. S2 The electron localization function of THO-C_3_N-1 monolayer.


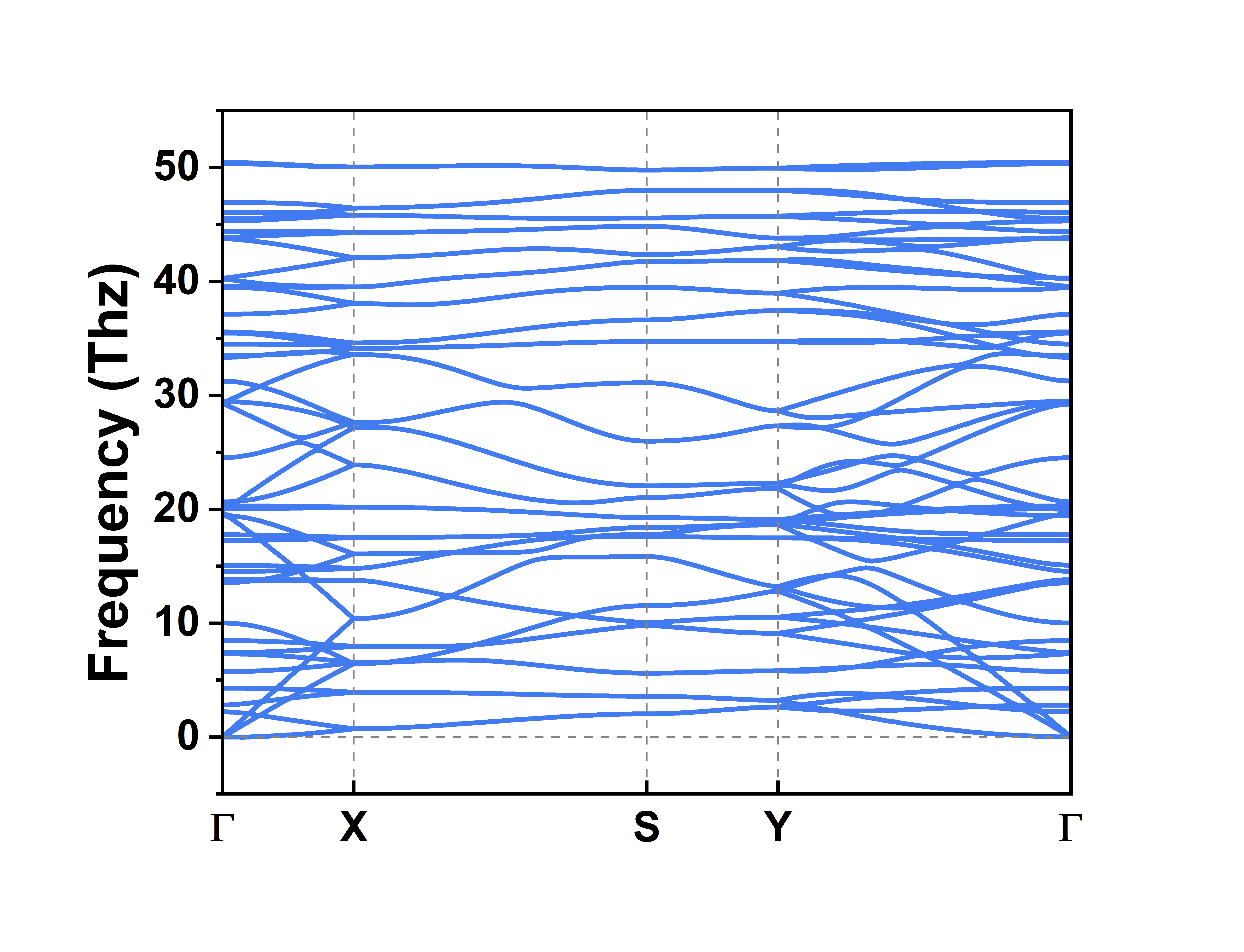


Fig. S3 Phonon dispersion relations of the THO-C_3_N-1 monolayer.

**
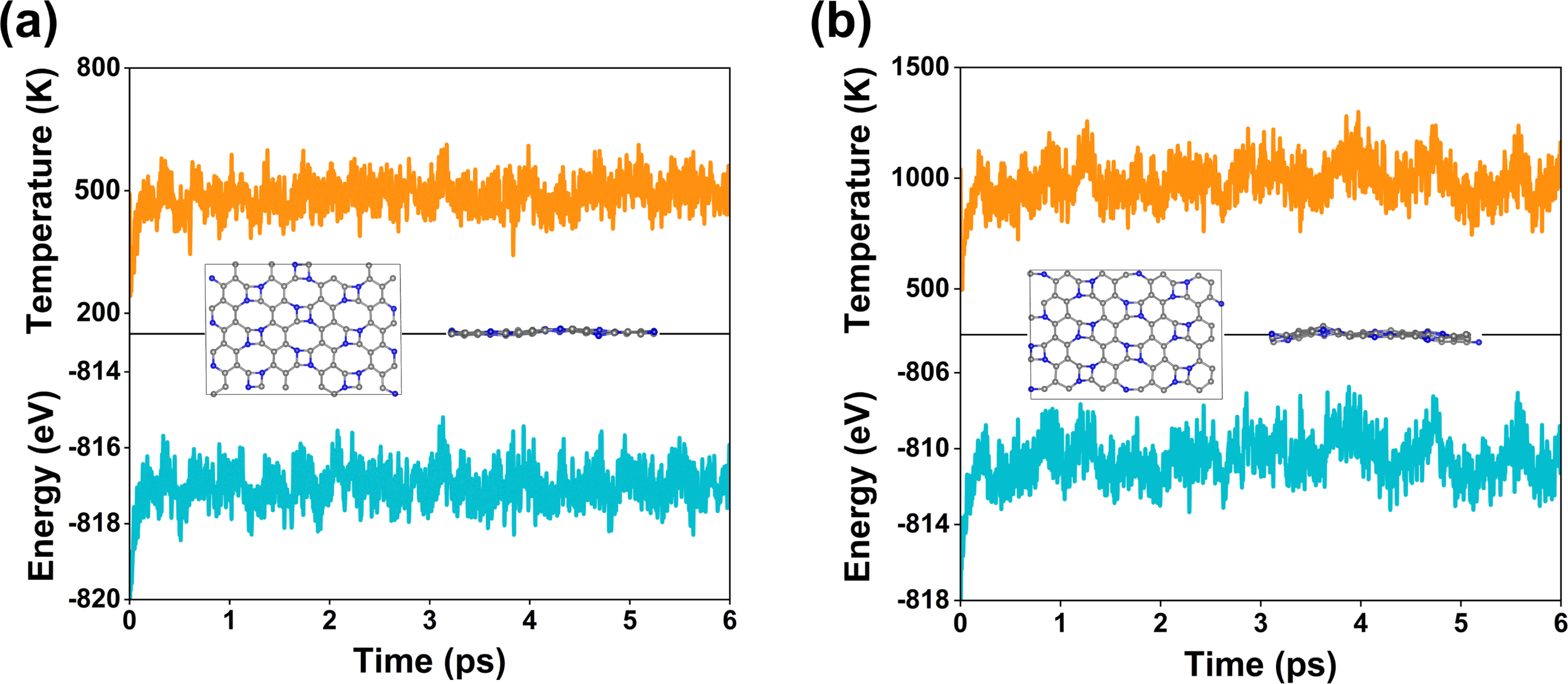
**

Fig. S4 Fluctuations of the temperature and total energy of THO-C_3_N-1 during the AIMD simulation at (a) 500 K, and (b) 1000 K. The insets are the simulated atomic configurations of THO-C_3_N-1 at the corresponding temperatures.


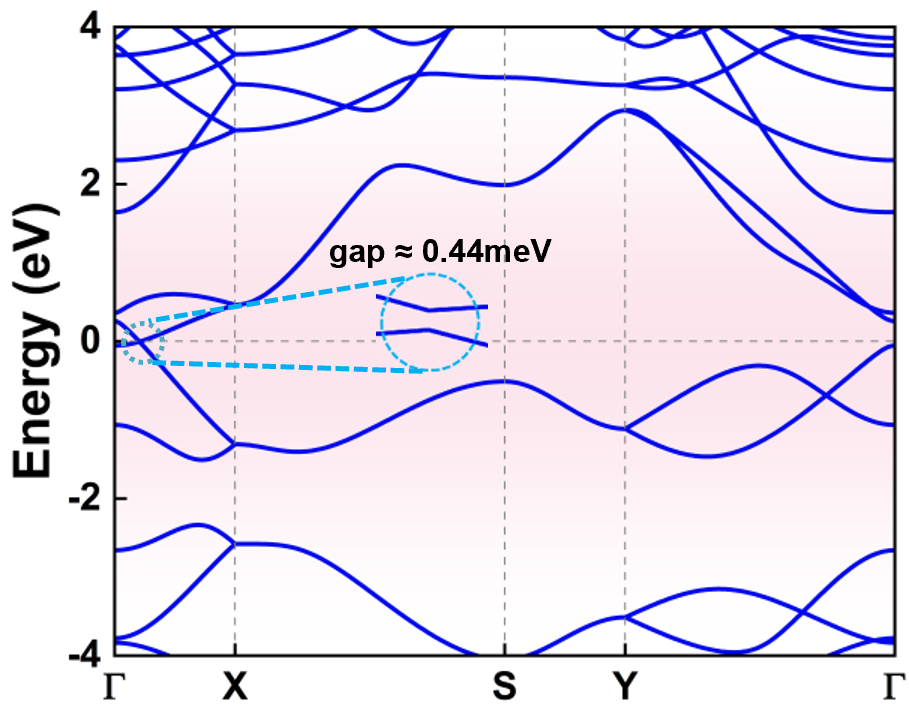


Fig. S5 The band structure of THO-C_3_N-1 with SOC.

**
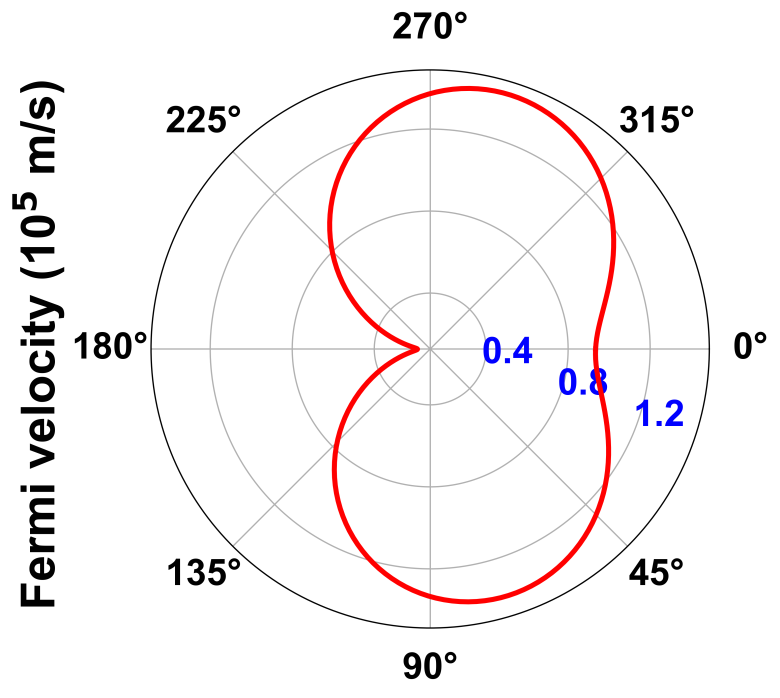
**

Fig. S6 Direction-dependent Fermi velocities of THO-C_3_N-1, calculated based on DFT results.

**
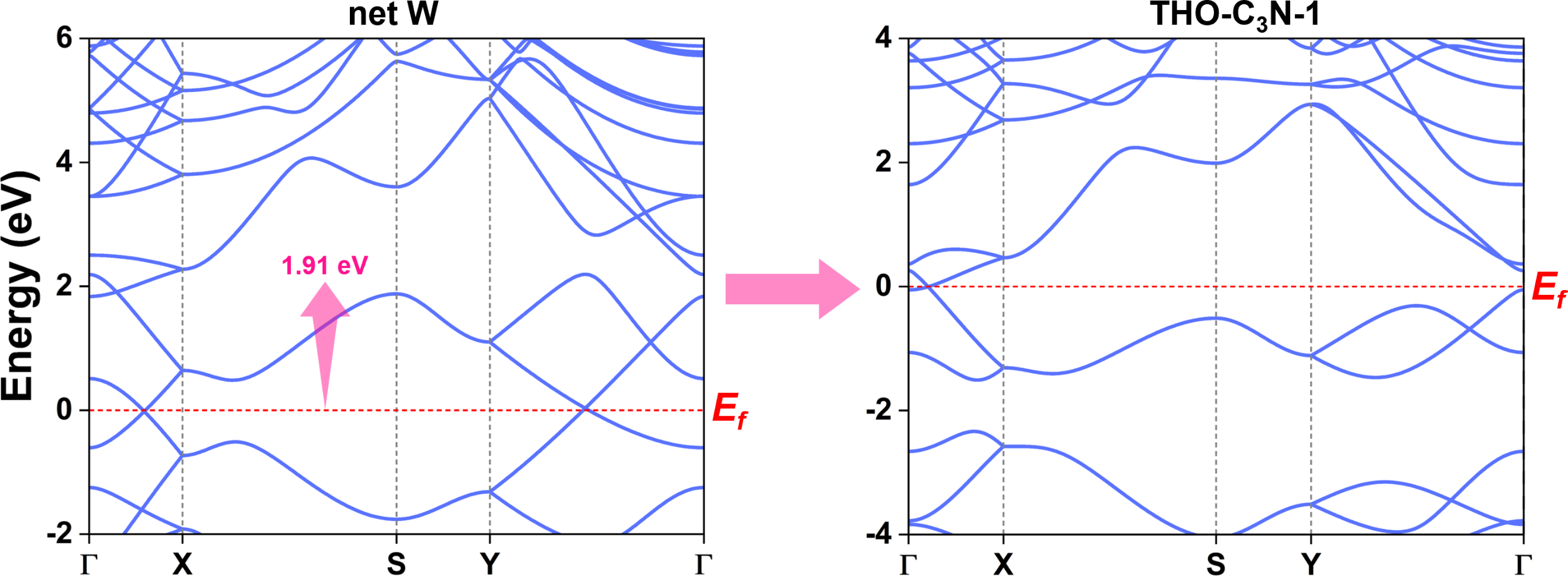
**

Fig. S7 N-doping induced the Fermi energy level shift and the change of band structure.

Table S1. The structure information of THO-C_3_N-2 and THO-C_3_N-3 monolayers.

| Materials | Space group | Lattice Parameters (Å, °) | Wyckoff Positions (fractional) | | | |
| --- | --- | --- | --- | --- | --- | --- |
| THO-C_3_N-2 | *Amm*2 | *a* = *b* = 5.49  *α* = *β* = 90.00  γ = 47.23 | Atoms | *x* | *y* | *z* |
|  |  |  | C1 (4e) | 0.93 | 0.32 | 0.50 |
|  |  |  | C2 (4e) | 0.31 | 0.33 | 0.50 |
|  |  |  | C3 (4e) | 0.93 | 0.66 | 0.50 |
|  |  |  | N (4e) | 0.31 | 0.67 | 0.50 |
| THO-C_3_N-3 | *Pbam* | *a* = 10.04  *b* = 4.44  *α* = *β* = γ = 90.00 | C1 (4h) | 0.81 | 0.34 | 0.50 |
|  |  |  | C2 (4h) | 0.43 | 0.66 | 0.50 |
|  |  |  | C3 (4h) | 0.43 | 0.34 | 0.50 |
|  |  |  | N (4h) | 0.69 | 0.16 | 0.50 |

**
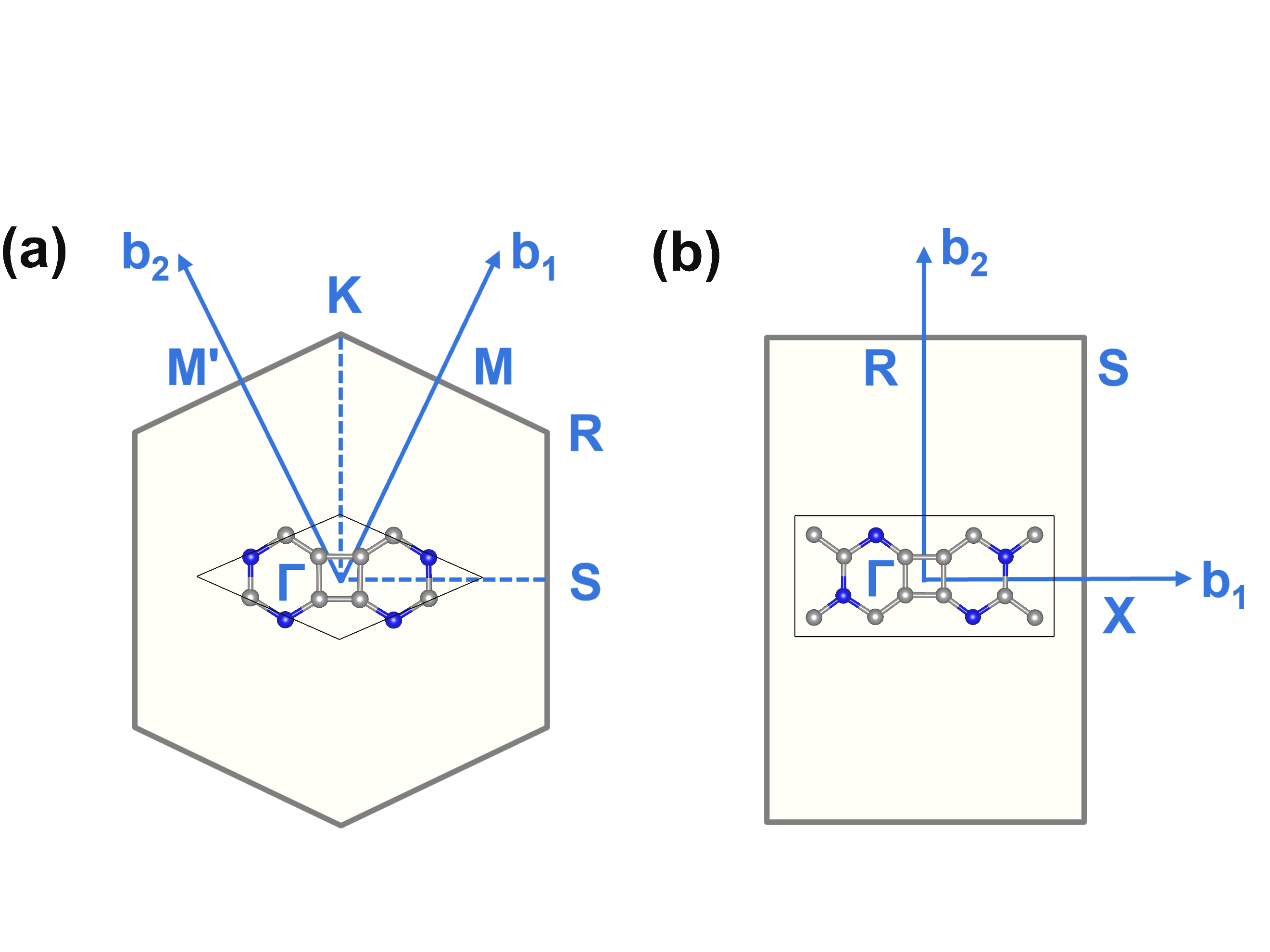
**

Fig. S8 The first Brillouin zone of (a) THO-C_3_N-2 and (b) THO-C_3_N-3 with high symmetry points and lines.


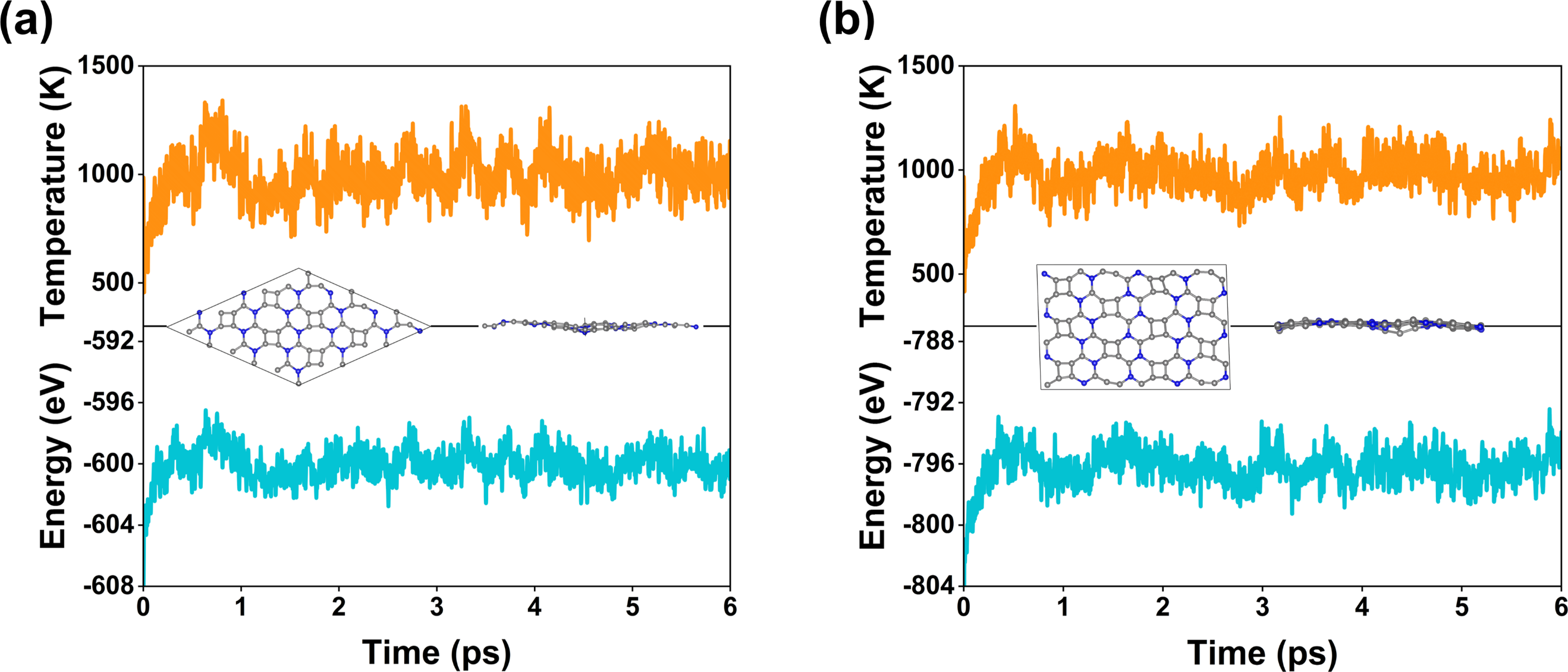


Fig. S9 AIMD simulations of (a) 3 × 3 × 1 THO-C_3_N-2 supercell and (b) 2 × 3 × 1 THO-C_3_N-3 supercell at 1000 K.

Table S2. The calculated in-plane elastic constants, Young’s modulus *E*(*θ*), in units of N/m, and the Poisson’s ratio *ν*(*θ*) for the THO-C_3_N-2, THO-C_3_N-3 and compare with three typical 2D materials.

| System | Elastic constants | | | | | *E*(*θ*) | | | *ν*(*θ*) | | |
| --- | --- | --- | --- | --- | --- | --- | --- | --- | --- | --- | --- |
|  | *C*_11_ | *C*_12_ | *C*_22_ | *C*_66_ | max | | min | max | | min |  |
| THO-C_3_N-2 | 359.75 | 80.62 | 270.16 | 128.10 | 335.70 | | 252.10 | 0.30 | | 0.20 |  |
| THO-C_3_N-3 | 360.68 | 80.31 | 271.36 | 129.57 | 336.91 | | 253.47 | 0.30 | | 0.20 |  |
| Graphene | 355.30 | 51.24 | – | – | 347.91 | | 347.91 | 0.14 | | 0.14 |  |
| biphenylene | 292.99 | 94.36 | 242.63 | 83.40 | 256.30 | | 212.24 | 0.39 | | 0.32 |  |
| C_3_N | 364.12 | 55.69 | – | – | 355.60 | | 355.60 | 0.15 | | 0.15 |  |


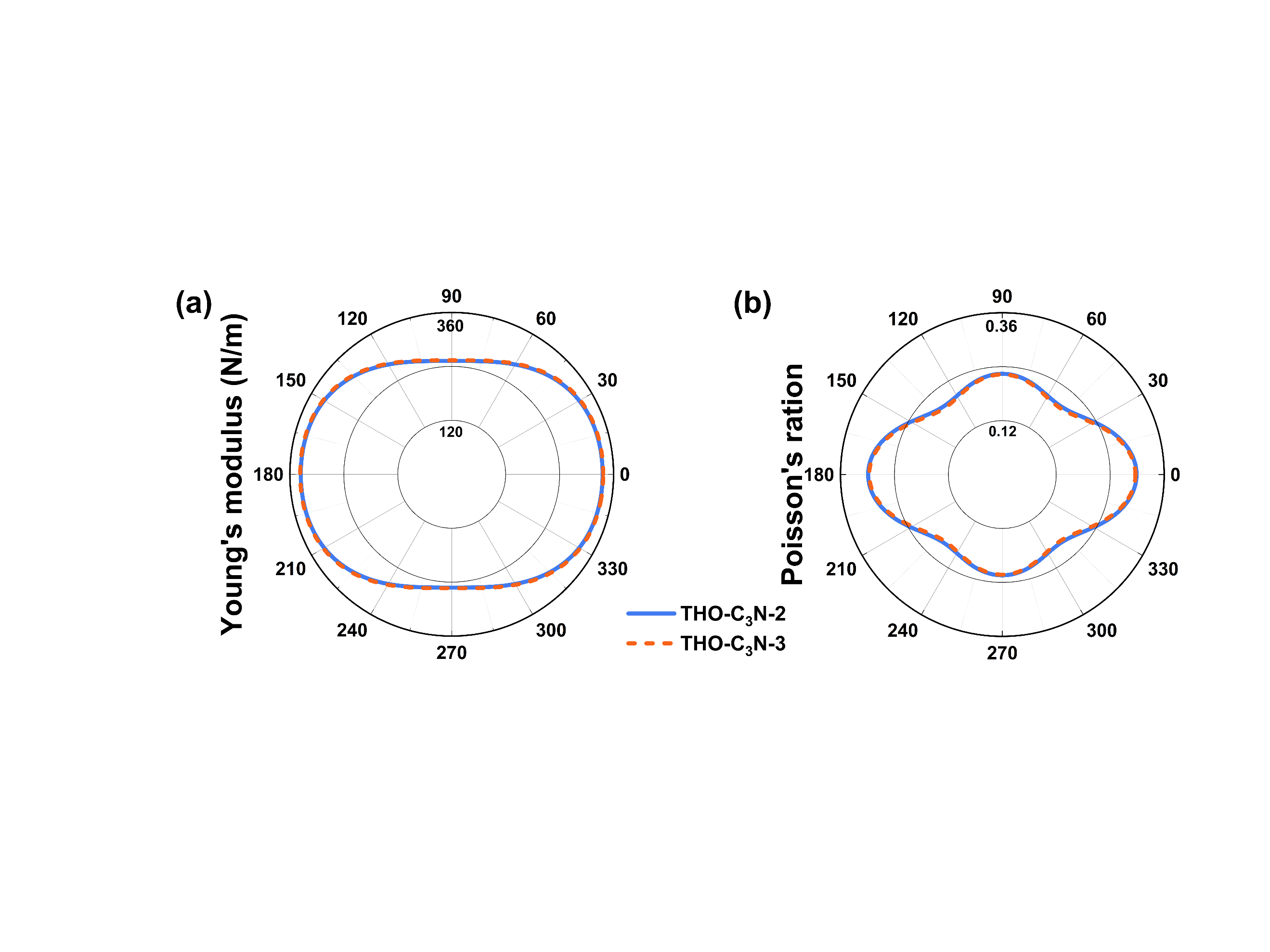


Fig. S10 Polar diagrams of the (a) Young’s modulus and (b) Poisson’s ratio for THO-C_3_N-2 and THO-C_3_N-3 monolayers.


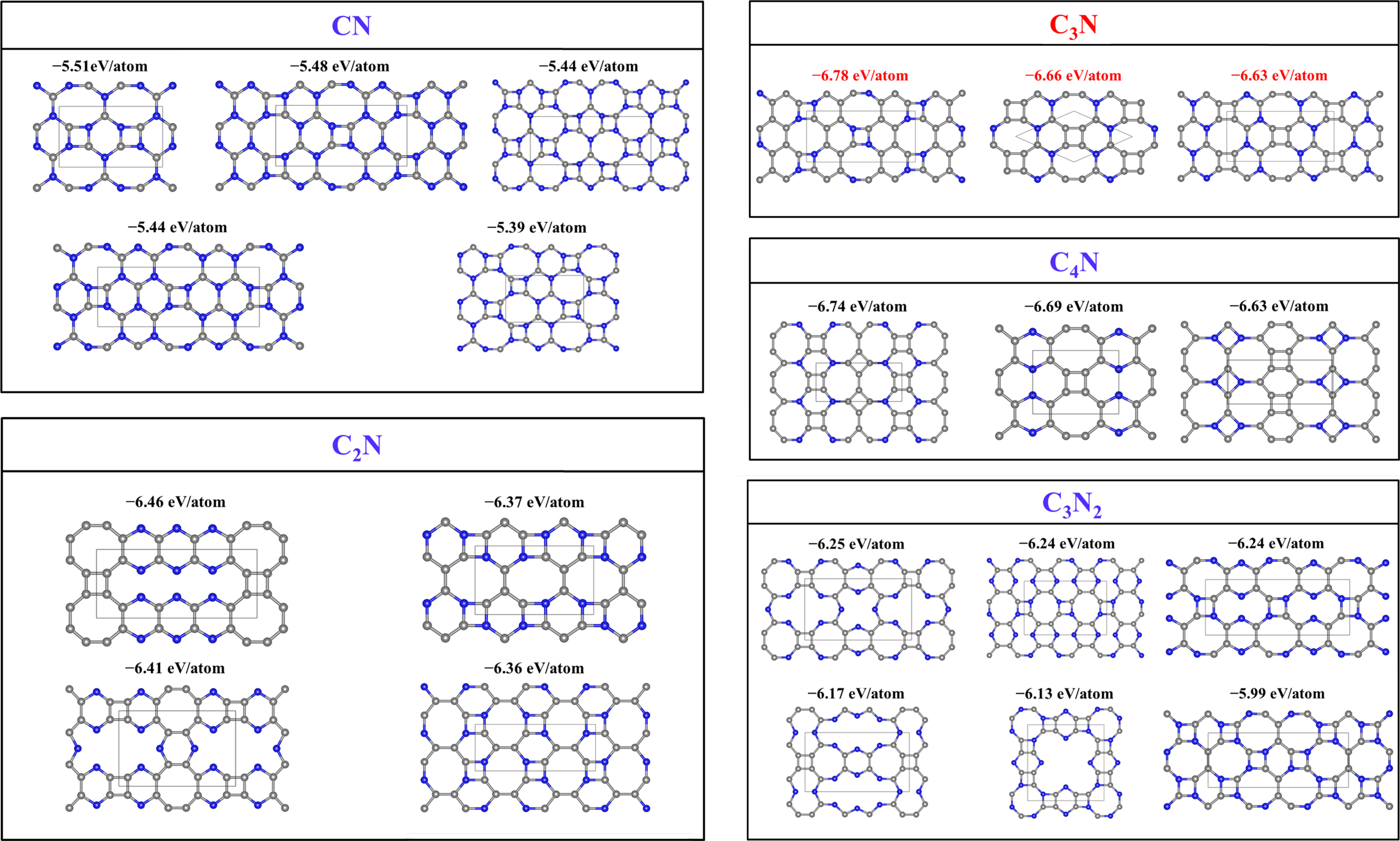


Fig. S11 Predicted 21 new 2D carbon nitrides constructed from four-, six-, and eight-membered rings, alongside their corresponding cohesive energies.


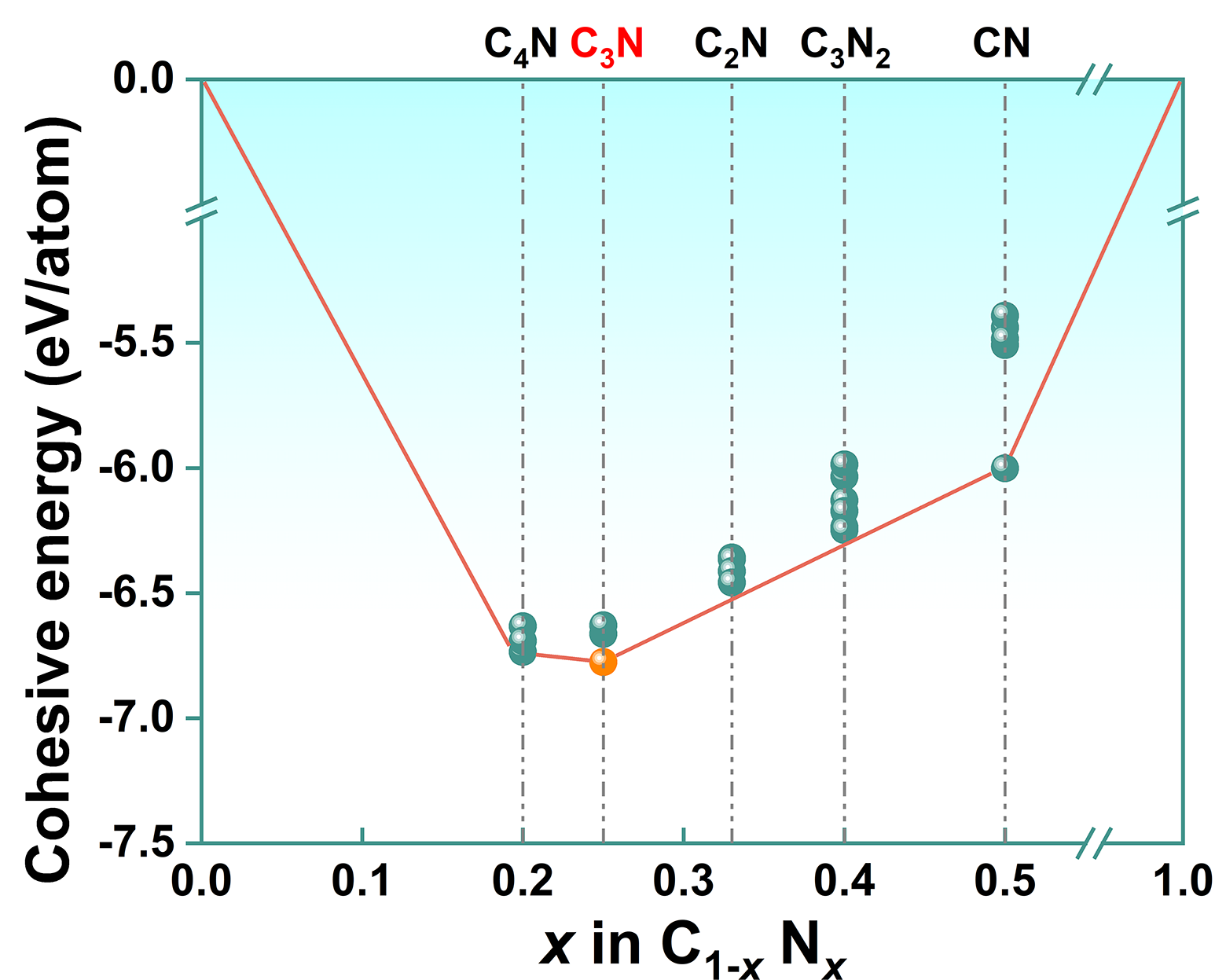


Fig. S12 Convex hull of theoretically predicted THO-C_3_N monolayers and other biphenylene-based carbon nitrides. The orange sphere marks THO-C_3_N-1.


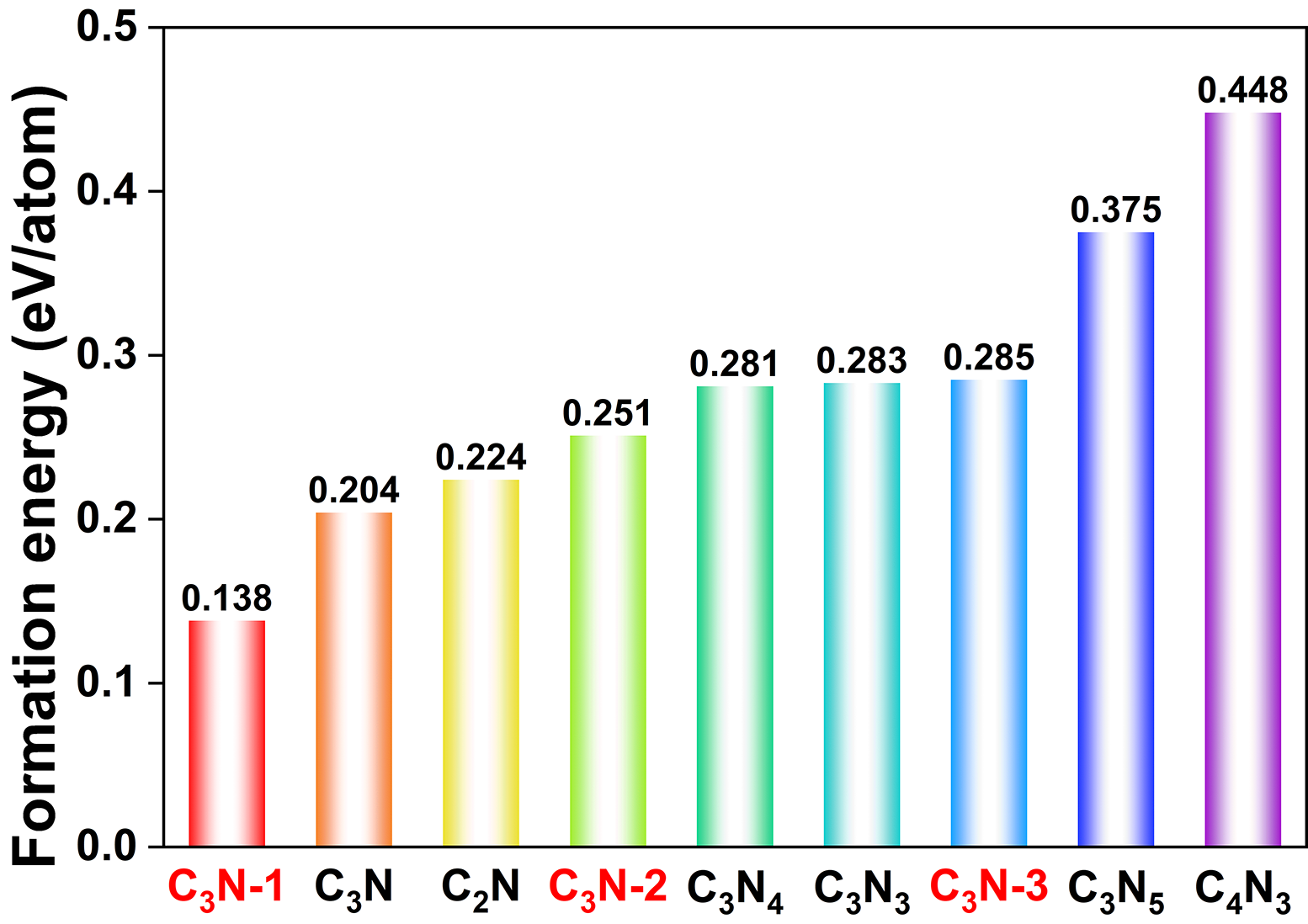


Fig. S13 Formation energy per atom of THO-C_3_N monolayers compared with synthesized 2D carbon nitrides.


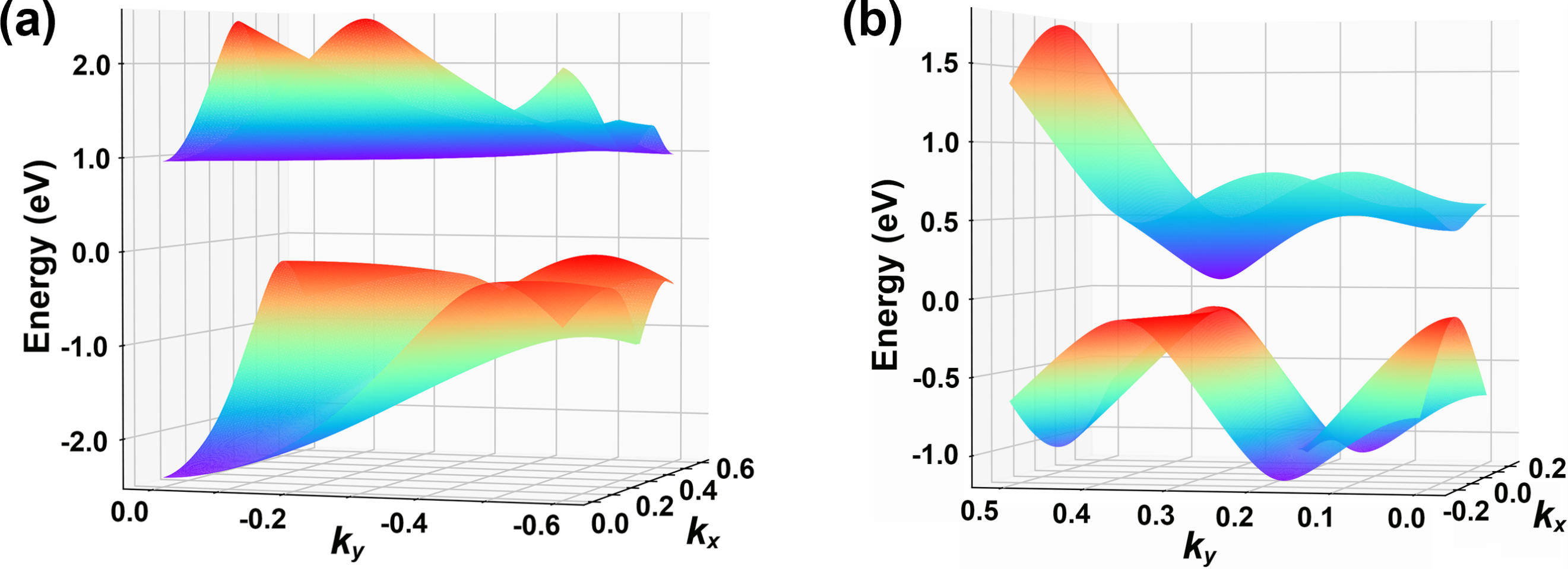


Fig. S14 Three-dimensional band structures of (a) THO-C_3_N-2 and (b) THO-C_3_N-3.


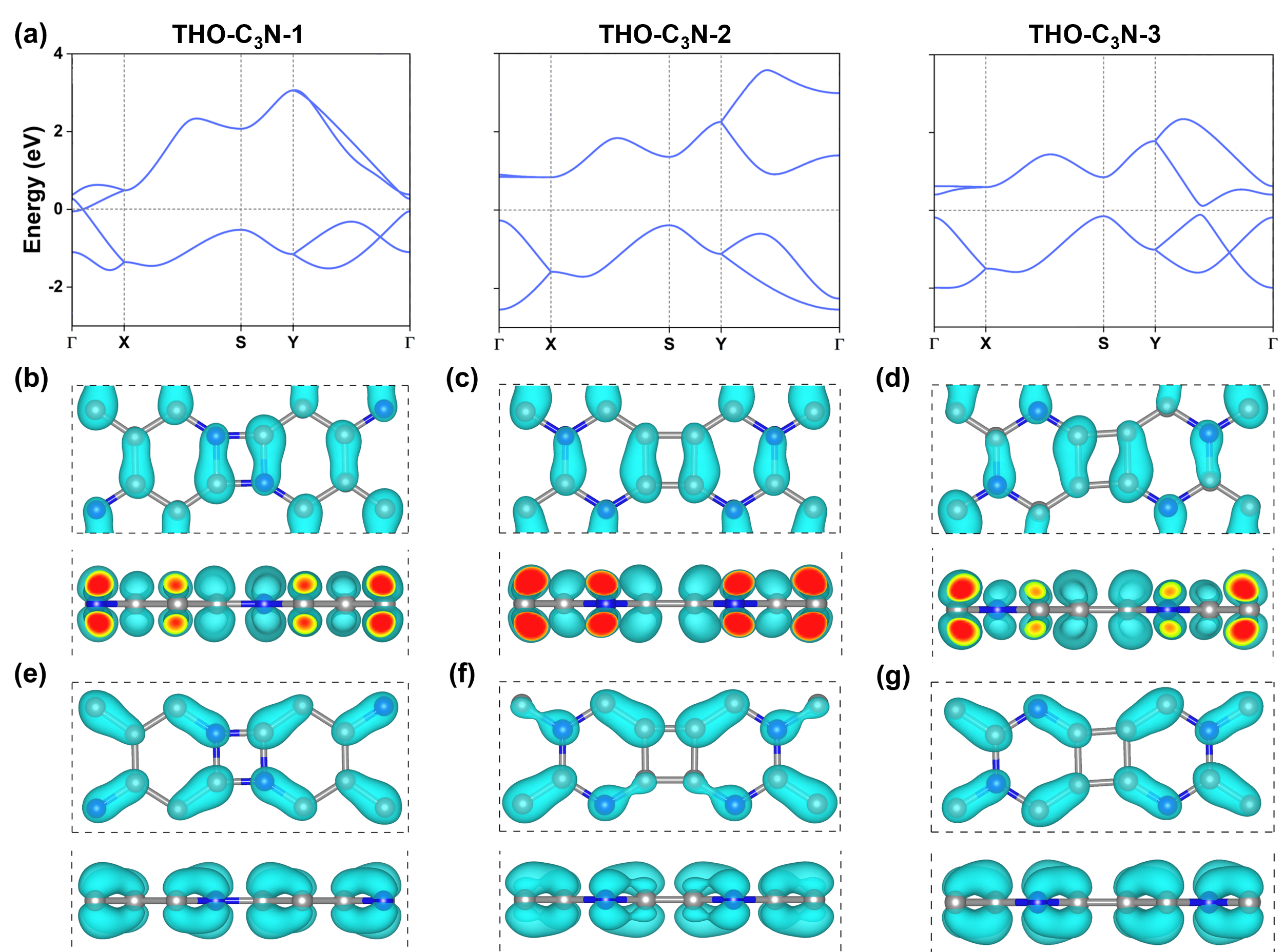


Fig. S15 (a) Band dispersions of four bands near the Fermi level in three THO-C_3_N monolayers. Top and side views of the band-decomposed charge density for the lowest conduction band at the *k*-point (0.103, 0, 0) of (b) THO-C_3_N-1, (c) THO-C_3_N-2, and (d) THO-C_3_N-3. Top and side views of the band-decomposed charge densities for the highest valence band at the *k*-point (0.103, 0, 0) of (e) THO-C_3_N-1, (f) THO-C_3_N-2, and (g) THO-C_3_N-3.


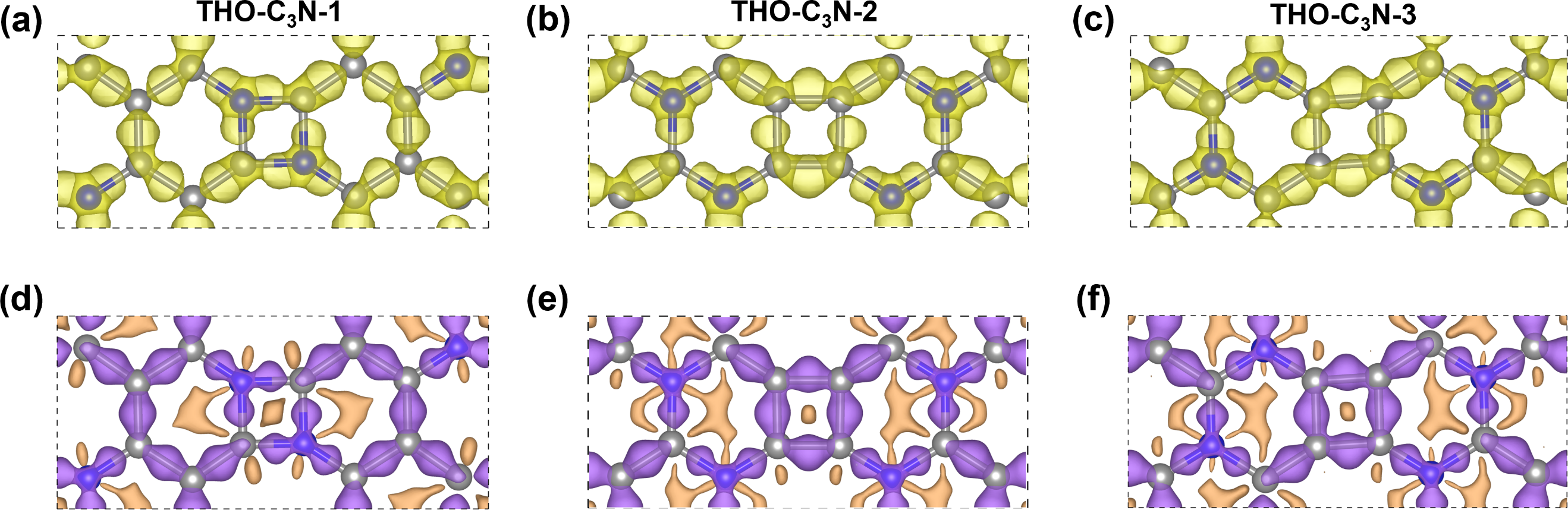


Fig. S16 The isosurface of the ELF of the (a) THO-C_3_N-1, (b) THO-C_3_N-2, and (c) THO-C_3_N-3 monolayers plotted with the isovalue of 0.7 *e* Å ^−3^. Deformation charge density of the (d) THO-C_3_N-1, (e) THO-C_3_N-2, and (f) THO-C_3_N-3 monolayers. Purple and brown refer to electron accumulation and depletion regions, respectively. The isovalue of deformation charge density is 0.12 *e* Å ^−3^.

Table S3. The Bader charge of three THO-C_3_N monolayers.

| Materials | Bader (\|*e*\|) | |
| --- | --- | --- |
|  | C | N |
| THO-C_3_N-1 | 0.41 | 1.23 |
| THO-C_3_N-2 | 0.40 | 1.20 |
| THO-C_3_N-3 | 0.42 | 1.26 |


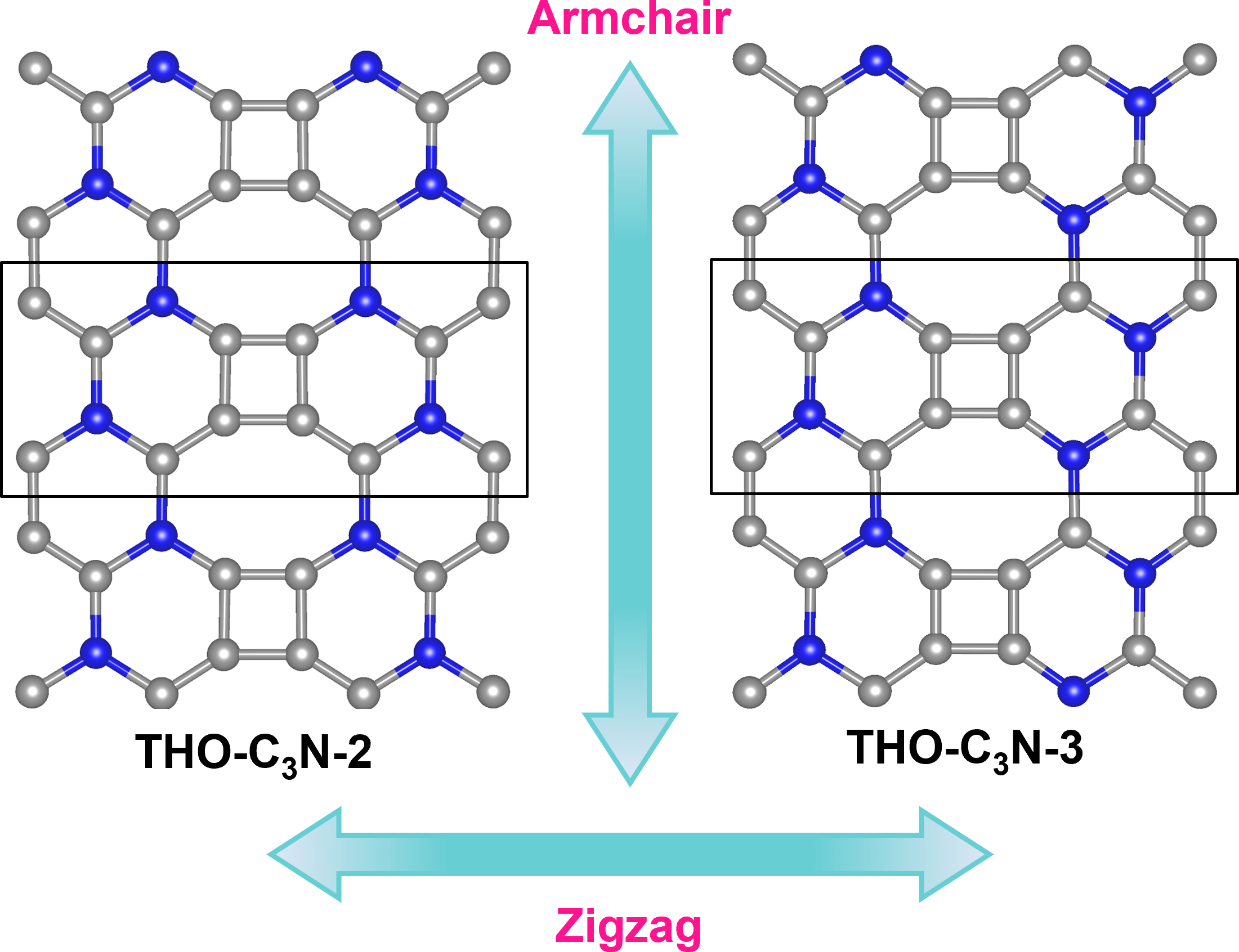


Fig. S17 Schematic illustrations of THO-C_3_N-2 and THO-C_3_N-3 monolayers along the zigzag and armchair directions, respectively.





Fig. S18 Temperature-dependent electron and hole mobilities along the *x* and *y* directions for the THO-C_3_N-2 at a carrier concentration of 1 × 10^16^ cm^−3^.





Fig. S19 Temperature-dependent electron and hole mobilities along the *x* and *y* direction for the THO-C_3_N-3 at a carrier concentration of 1 × 10^16^ cm^−3^.


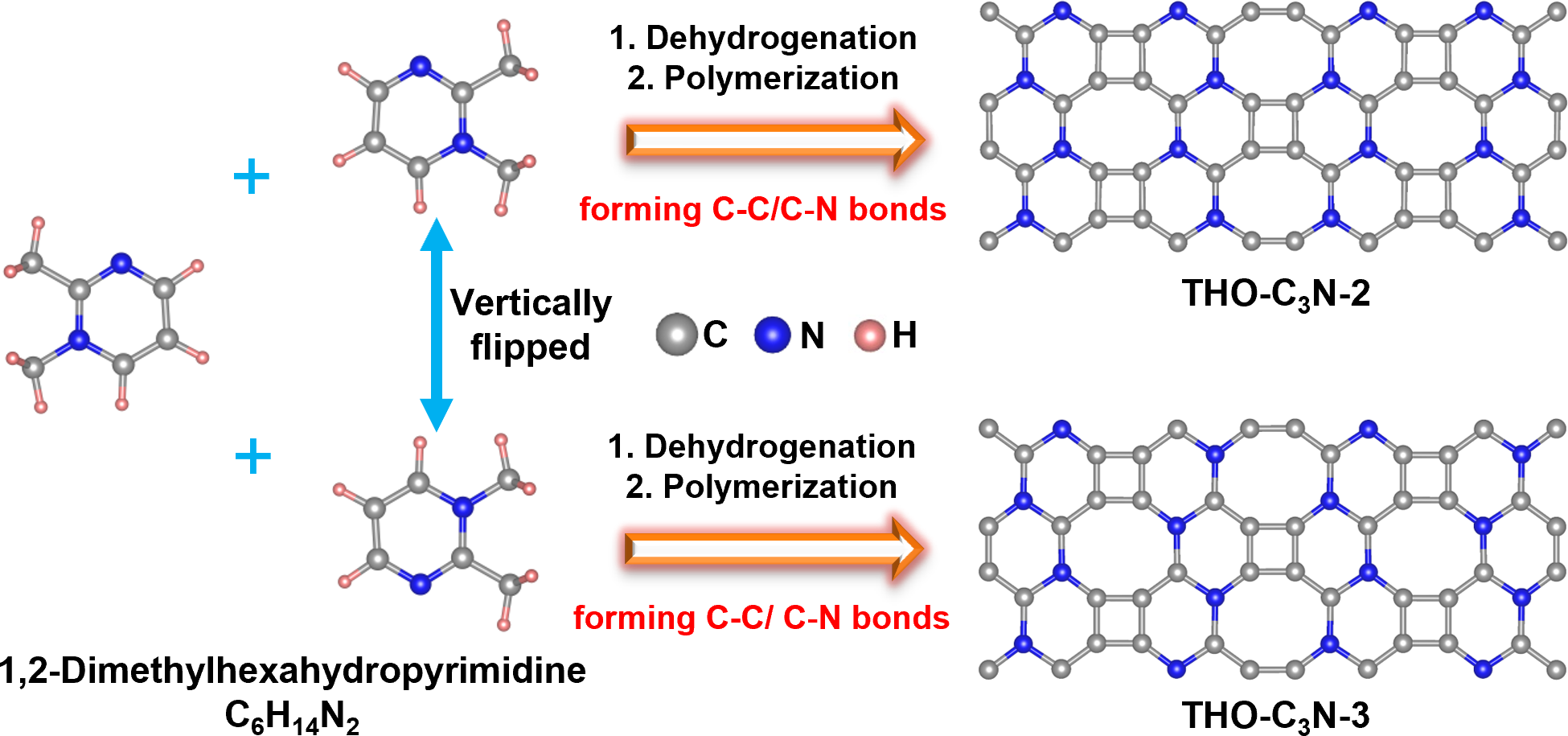


Fig. S20 Possible synthetic pathway of THO-C_3_N-2 and THO-C_3_N-3.


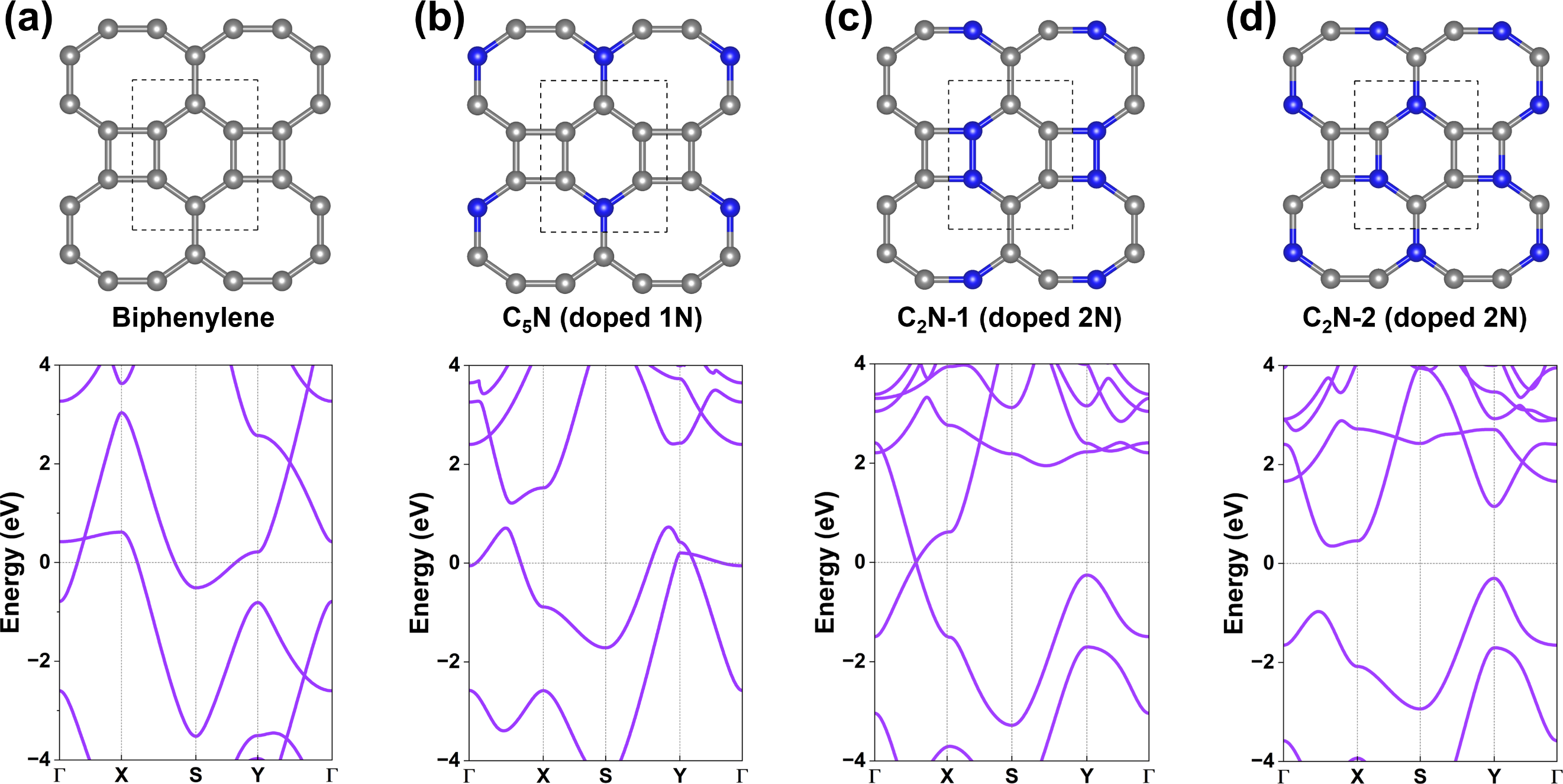


Fig. S21. Crystal structures and corresponding band structures of BPN under different N-doping configurations. The unit cell is indicated by a dashed rectangle.





Fig. S22 Phonon dispersion of THO-C_3_N-2 for various supercell sizes and the variation of the POP frequency with supercell size.





Fig. S23 Phonon dispersion of THO-C_3_N-3 for various supercell sizes and the variation of the POP frequency with supercell size.
